# Rates of contributory de novo mutation in high and low-risk autism families

**DOI:** 10.1038/s42003-021-02533-z

**Published:** 2021-09-01

**Authors:** Seungtai Yoon, Adriana Munoz, Boris Yamrom, Yoon-ha Lee, Peter Andrews, Steven Marks, Zihua Wang, Catherine Reeves, Lara Winterkorn, Abba M. Krieger, Andreas Buja, Kith Pradhan, Michael Ronemus, Kristin K. Baldwin, Dan Levy, Michael Wigler, Ivan Iossifov

**Affiliations:** 1 Cold Spring Harbor Laboratory, Cold Spring Harbor, New York, NY USA; 2 New York Genome Center, New York, NY USA; 3 Statistics Department, The Wharton School, University of Pennsylvania, Philadelphia, PA USA; 4 Department of Medicine, Albert Einstein College of Medicine, Montefiore Medical Center, Bronx, NY USA; 5 Department of Neuroscience, The Scripps Research Institute, La Jolla, CA USA; 6 Department of Genetics and Development, Columbia University, New York, NY USA

**Keywords:** Next-generation sequencing, Autism spectrum disorders, Autism spectrum disorders

## Abstract

Autism arises in high and low-risk families. *De novo* mutation contributes to autism incidence in low-risk families as there is a higher incidence in the affected of the simplex families than in their unaffected siblings. But the extent of contribution in low-risk families cannot be determined solely from simplex families as they are a mixture of low and high-risk. The rate of de novo mutation in nearly pure populations of high-risk families, the multiplex families, has not previously been rigorously determined. Moreover, rates of de novo mutation have been underestimated from studies based on low resolution microarrays and whole exome sequencing. Here we report on findings from whole genome sequence (WGS) of both simplex families from the Simons Simplex Collection (SSC) and multiplex families from the Autism Genetic Resource Exchange (AGRE). After removing the multiplex samples with excessive cell-line genetic drift, we find that the contribution of de novo mutation in multiplex is significantly smaller than the contribution in simplex. We use WGS to provide high resolution CNV profiles and to analyze more than coding regions, and revise upward the rate in simplex autism due to an excess of de novo events targeting introns. Based on this study, we now estimate that de novo events contribute to 52–67% of cases of autism arising from low risk families, and 30–39% of cases of all autism.

## Abstract (web summary)

Yoon, Munoz, et al. investigate the rate of de novo coding and non-coding variants in families with high- and low-risk for autism using whole-genome sequence data from collections of families with autism. They demonstrate that de novo intronic variants increase the risk of autism, that the contribution of de novo variants is significantly larger in low-risk families, and that de novo variants contribute to 30-39% of cases of all autism.

## Introduction

Autism is a neurodevelopmental disorder with a strong genetic component. This was first inferred by the greater concordance of monozygotic twins than dizygotic twins and an increase in risk to the siblings of an affected child. The importance of genetics was later directly demonstrated by finding a higher incidence of de novo gene disruption in affected than unaffected siblings within families with only a single affected offspring (simplex families)^1–12^. This observation, plus the higher incidence of autism in males than females (3:1), the observed rate of ~25% concordance for boy siblings, and the nearly 50% incidence of autism in the third born male child of multiplex families^13^ led to what we call the “unified genetic theory for sporadic and inherited autism” in 2007. In this theory we conjectured two risk classes of families. We proposed that highly penetrant de novo mutations contribute to most low-risk families and that transmission from a carrier parent contributed to autism in most high-risk families. We used data on sibling risk and brood size to estimate that roughly half of autism arises from low-risk families and half from high-risk families^14,15^.

We still lack a clear demonstration that low and high-risk families are truly distinct in their genetic causation. The role of de novo mutations has been firmly demonstrated in collections of simplex families that would be expected to contain mainly low-risk families. On the other hand, the incidence of de novo mutation in multiplex families, which would be expected to be almost exclusively high-risk, has been equivocal^5,7^, and it is known that common variation plays a substantial role in autism heritability^16,17^. Unlike the simplex collections, largely drawn from blood, many of the multiplex collections are drawn from EBV-immortalized lymphoid cells maintained in culture. These, due to oligo-clonality and/or cell-line genetic drift, have an excess of somatic artifacts, rendering measurement of de novo rates problematic. We have sought to compare the rates in simplex and multiplex collections by controlling for drift in the latter. Our results of low rates in multiplex confirm a recent study of the same population^18^.

Basing the contribution of de novo variants in simplex collections solely on large copy number events (CNV) and the likely-gene damaging (LGD) coding mutations will lead to underestimates. A great deal of uncertainty still exists for the role of missense mutations, the full contribution from CNVs and other structural rearrangements, and only preliminary attempts have been made in the non-coding space^19–25^. Thus, quantifying the role of de novo mutation in simplex families is still an open problem.

In this paper we used the newly generated whole genome sequencing (WGS) data from large multiplex (AGRE) and large simplex (SSC) family collections to compare the contributions of de novo variants in low and high-risk families and to evaluate the role of de novo intronic variants in SSC. We consider large scale copy number events, small indels, and substitutions. First, however, we identify the AGRE multiplex samples with extensive somatic genomic drift by the presence of two hallmarks: (1) excess single nucleotide variations throughout the entire genome; and (2) a preponderance of variants with unexpected allele ratios. Removing these samples, we then measure rates of de novo events in the remainder. From this new data, we make better estimates of contribution of de novo mutation in collections of simplex and multiplex families. Our analysis points to a substantial contribution of de novo variants in introns. From our results we update estimates of the contribution of de novo events in high and low-risk families and in autism overall.

## Results

### Comparing de novo rates in multiplex and simplex families

#### Filtering multiplex samples with excessive cell-line genetic drift

**Fig. 1:**
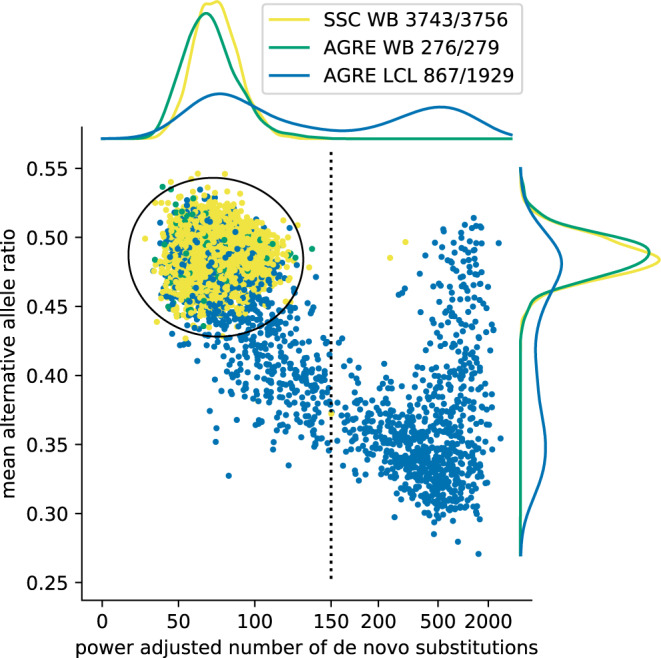
Cell-line genetic drift filter. Each dot represents a data point from one individual child. Individuals from AGRE for which DNA was extracted from LCLs (AGRE LCL) are colored blue, individuals from SSC, for all of which DNA was extracted from whole blood (SSC WB) are colored yellow, and individuals from AGRE extracted from whole-blood DNA (AGRE WB), are colored green. We adjusted the number of observed *de novo* substitutions based on the power for detection of *de novo* SNVs estimated separately for every child (see Materials and Methods for description of procedure for power estimation and Supplementary Figure 2). We set the adjusted number of *de novo* substitutions to the observed number of *de novo* substitutions divided by the estimated power and show it on the X-axis in linear scale for numbers smaller than 150 (the vertical dashed line) and in log-scale for larger numbers. For every child, we assigned the mean alternative allele ratio based on the alternative allele ratios (defined as the proportion of the sequencing reads converting the position that support the alternative allele) for each of the *de novo* substitutions identified in the child. The mean alternative allele ratios are shown on the Y-axis. Density plots in the top and right sides show marginal distributions of corresponding cohorts by color. We modeled the WB data from SSC and AGRE as a two-dimensional Gaussian distribution over the power adjusted number of *de novo* substitutions and the mean alternative allele ratio and we established an ellipse (shown in black) that would include 99.9% of the Gaussian distribution density. Children within the ellipse are considered free of cell-line genomic drift. We show in the inset the total number of children in each of the three groups together with the number of children that were determined to be free of cell-line genetic drift.

Some of the AGRE DNAs used for WGS were extracted from cultured lymphoblastoid cell lines (LCL), and some from whole blood (WB). In contrast all of the SSC DNA was extracted from whole blood (WB). Samples from LCL pose a risk: without care, the large number of events acquired during cell culture or immortalization will be mistaken for germline mutation. These artifacts are made evident in the AGRE by examining the frequency and allele ratios of the substitutions observed in the children but not in either parent (Fig. 1). For each child-sample we display in a scatter plot its number of “acquired” substitutions adjusted for coverage (X-axis) and the mean ratio of heterozygosity for those substitutions (Y-axis). The distributions of these two properties are shown in the margins for the LCL from AGRE (blue), the WB from AGRE (green), and the WB from the SSC (yellow). Clearly the WB data from AGRE and SSC show similar distributions. On the other hand, the LCL data is multiphasic, with one phase in agreement with WB.

To identify the usable samples, we modeled the WB data as a two-dimensional Gaussian distribution on the variables of allele count and ratio (see Fig. 1 and Supplementary Data 1). We then established an ellipse that would exclude 0.1% of the Gaussian distribution. This ellipse empirically excludes about 0.3% (13 out of 3756) of the WB samples. We consider the samples in the ellipse to be free of cell-line genetic drift. We then apply the same filter to the LCL of the AGRE, and thereby exclude an excess of 50% of those (1062 out of 1929). We note that our method of filtering agrees very well with the machine learning method of Ruzzo, et al.^18^, even though the latter is conditioned on the variant and not the sample. Consistent with our analysis based on substitutions, the same samples with detected cell-line genetic drift have an excess of acquired CNVs (Supplementary Fig. 1 top two panels), and many of these CNVs do not have integer copy number (Supplementary Fig. 1 bottom two panels).

#### Comparing AGRE and SSC for small scale de novo mutations in genes

For the rest of the analysis, we selected three groups from the trios passing the sample cell-line genetic drift filters: 1,874 unaffected children from SSC; 1,869 affected children from SSC; and 1,107 affected children from AGRE. There were not enough of the unaffected siblings from AGRE for meaningful analysis. In selected children, we identified small scale de novo events, both substitutions and small indels, using previously published methods^2,11^ (see Materials and Methods, Supplementary Data 2 and Supplementary Data 3 for the list of all small scale de novo variants identified). These small events were then divided into six likely functional classes and counted: LGD (likely gene disrupting, consisting of nonsense, frameshift, or splice site mutations); synonymous (SYN); intronic substitutions (ISB) and small indels (IID); and intergenic substitutions (IGSB) and indels (IGID). We consider the SYN, IGSB, and IGID as free of any bias due to affected status because they will be largely without functional consequence. We do not consider in this paper missense substitutions, because their functional significance is too difficult to assess. Moreover, we exclude from consideration events on the X-chromosome.

We are interested in determining the extent to which LGD incidence is increased due to the affected status of the child within its family type. These must be adjusted for coverage, parental age, and possible cell-line genetic drift. The three groups of children were not identical in (1) depth of WGS coverage and (2) distribution of the ages of their parents (see Supplementary Fig. 2). Moreover, there is the possibility that (3) we have not completely filtered all cell line drift. These three factors clearly influence the rate of the observed de novo variants. To control for these variable rate factors, we used SYN mutations. SYN and LGDs are both located in protein coding regions and are likely to be affected similarly by parental ages, sequence coverage, and drift. We thus use de novo SYN incidence as a normalization factor for the comparison of LGD incidence in different populations, as we and others have done in the past^26^.

We outline here the method we use to compare the incidence of the subject class of LGD de novo variants in a group of SSC unaffected and a group of either AGRE or SSC affected using a normalization by of de novo SYN variants. We assume that the subject class (LGD) may be biased based of the affected status while the normalization class (SYN) is not, but that both classes are equally influenced by the covariates parental ages, sequence coverage and cell-line genetic drift. We are interested in two measures quantifying the role of the subject class in the affected group, the ascertainment differential (AD) and percent contributory (PC). The AD measures the excess of events above the estimate of expected number of observations of that subject class given the null hypothesis that these mutations had not contributed to the diagnosis. We compute the expected number of observations in the affected by this equation:

1 $${{{{{\rm{ES}}}}}}.{{{{{\rm{a}}}}}}={{{{{\rm{S}}}}}}.{{{{{\rm{u}}}}}}\;^*\; ({{{{{\rm{N}}}}}}.{{{{{\rm{a}}}}}}/{{{{{\rm{N}}}}}}.{{{{{\rm{u}}}}}})$$

where ES.a is the expected number of observations of the subject class in the affected group, S.u is the actual observed number of that class for the unaffected group, N.a is the observed number of the normalization class in the affected, and N.u is the observed number of that class in unaffected. The ascertainment differential (AD) for the affected group is defined as the difference between the number of observed events and the expected number, divided by the number of children in the group, and expressed as a percentage of children:

2 $${{{{{\rm{AD}}}}}}=100\;^*\; ({{{{{\rm{S}}}}}}.{{{{{\rm{a}}}}}} - {{{{{\rm{ES}}}}}}.{{{{{\rm{a}}}}}})/{{{{{\rm{C}}}}}}.{{{{{\rm{a}}}}}},$$

where the S.a is the observed number of the subject class in the affected group and C.a is the number of affected children in that group. We interpret AD as the percent of affected children that were diagnosed with autism due to a contribution from the given subject class, given the assumption that the expected number of contributory events per child is smaller than 1. We define the second measure, the percent contributory (PC), with this formula:

3 $${{{{{\rm{PC}}}}}}=100\;^*\; ({{{{{\rm{S}}}}}}.{{{{{\rm{a}}}}}} - {{{{{\rm{ES}}}}}}.{{{{{\rm{a}}}}}})/{{{{{\rm{S}}}}}}.{{{{{\rm{a}}}}}}.$$

We interpret PC as the percent of the subject class events in the affected group that have contributed to the disorder.

We compute a *p*-value of the AD under the null hypothesis: the subject class does not contribute to the disorder. This *p*-value is determined empirically as the likelihood of the observed AD arising from the data when we permute the labels “affected” and “unaffected”. Specifically, we measure the probability to achieve AD larger or equal to the observed AD under normal distribution fitted to ADs derived from 1,000 trials permuting the affected and unaffected, keeping the numbers of each group fixed. We also compute confidence intervals (CI) for the AD and the PC using 1,000 bootstrap iterations, sampling with repetition the same number of children from the set of the affected and unaffected.

With these methods we examined de novo LGD, a class of variant that is known to contribute to autism in simplex families. While most of the previous studies of ascertainment bias in this class was based on whole exome sequencing of the SSC, in this instance we have WGS from both the SSC and AGRE.

The AD and PC for the de novo LGDs in affected children from SSC are 6.48% (CI: 3.85 to 8.80) and 42.8% (CI: 27.8 to 54.3) respectively with *p*-value 8 × 10^−7^ (Table 1). These results are consistent with the well-established causal role of de novo LGDs in simplex autism^2,8,10,12,27^, and are comparable to previous results from WES. The AD and PC for the affected children for the multiplex families in AGRE are 1.42% (CI: −1.25 to 4.15) and 13.6% (CI: −13.4 to 35.1) with *p*-value of 0.14.

**Table 1:** Role of *de novo* LGDs in simplex and multiplex autism.

	SSC unaffected		Affected						
Group	Synonymous number	LGD number	Synonymous number	LGD number	Expected LGD number	Delta	*p-*value	AD	PC
SSC affected	484	157	499	283	161.9	121.1	8 × 10^−07^	6.48% (3.85–8.80)	42.8% (27.8–54.3)
AGRE affected			309	116	100.2	15.8	0.14	1.42% (−1.25–4.15)	13.6% (−13.4–35.1)

The table shows the numbers of *de novo* LGDs (“LGD number” columns) identified in 1,874 unaffected children from SSC, 1,869 affected children from SSC and 1,107 affected children the AGRE collection together with the number of *de novo* synonymous mutations (“synonymous number” columns), used for normalization, identified in the same children. The table also shows the expected number of *de novo* LGDs (“expected LGD number” column) in the two affected groups under the null model that the incidence of the such variants in the affected children is the same as the incidence in the unaffected children. The rest of the columns quantify the causal role of the *de novo* LGDs in the affected children from the simplex SSC and the multiplex AGRE collections. The “delta” column shows the excess of the *de novo* LGDs in the two affected groups. The “*p*-value” is the probability of achieving a delta larger or equal to the observed one under the null model, computed through permutation of the affect status. The “AD” column shows the ascertainment differential or our estimate of the percent of the affected children that have autism in part due to a *de novo* LGD; the “PC” column shows the percent contributory statistic that is the percent of the *de novo* LGDs identified in the affected children that contributed to the disorder. Finally, the table shows the 95% confidence interval for the AD and PC statistics computed by bootstrap. See the Results section for detailed description of the methods used.

These results suggest that de novo LGDs contribute to autism in the high-risk multiplex families, but the contribution is most likely less than a quarter of the contribution of such variants in simplex families. The AD measures in the two affected groups are statistically different, with *p*-value of 0.001 computed by directly comparing the two groups of affected.

#### Comparing AGRE and SSC for large scale de novo mutations

We developed a pipeline for detection of de novo CNV events which was based on two different methods and a set of stringent filters to avoid false-positive calls (e.g. cryptic transmission). The pipeline is described in the Materials and Methods and Supplementary Note 1 and all detected events are listed in Supplementary Data 4. As we did for the small-scale events, we compared the affected children in SSC and AGRE to the unaffected in SSC and restricted the analysis only to the children that passed the sample cell-line genetic drift filters. As discussed above the three groups of children differ in the ages of their parents and in the sequence coverage. We ignored the difference of parental ages because there is no well-documented relationship between parental ages and rates of de novo CNV. Moreover, we see no evidence for such relationship in our data. We examined the effects of coverage in the three groups on the power of our pipeline to detect de novo CNVs by simulating artificial CNVs of various sizes (see legends and Materials and Methods). The results (Supplementary Fig. 4) show that the power is the same for the affected and unaffected children from SSC across all CNV sizes (from 1 kb to 100 kb). But the power to detect small (<4 kb) CNVs in the data from the AGRE affected is diminished. In comparing the SSC to AGRE families, therefore, we ignored the CNVs smaller than 4 kb. We simplified the definitions of AD and PC to avoid the normalization for coverage and parental ages by using the number of children instead of the number of de novo variants of a normalization class.

**Fig. 2:**
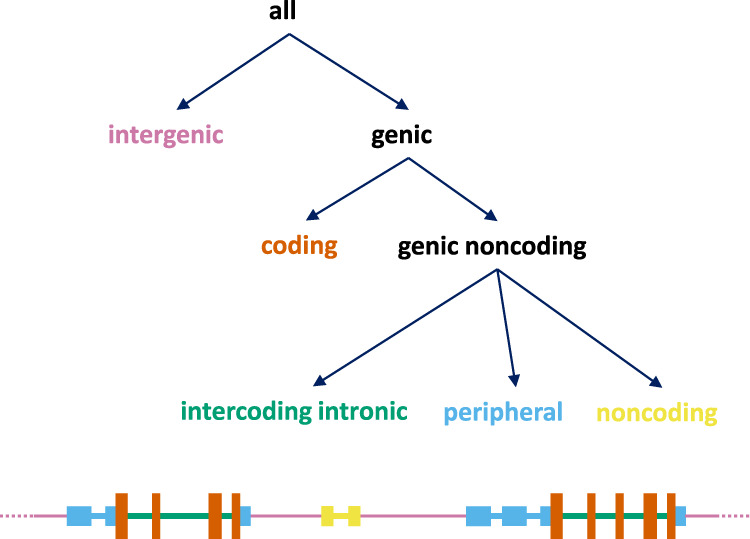
Event types. We assign a label ‘intergenic’ (purple) to any event that falls between transcripts; a label ‘coding’ (brown) is assigned to events that affect coding exons; a label ‘intercoding intronic’ (green) is assigned to events that affect an intron splitting two coding exons; a label ‘peripheral’ (blue) is assigned to events that affect coding transcripts but are neither coding nor ‘intercoding intronic’; a label ‘noncoding’ (yellow) is assigned to events that affect noncoding transcripts. The peripheral events affect the untranslated regions of the coding transcripts (UTRs) or the introns that split UTRs. We use RefSeq transcript models downloaded from the UCSC genome browser to find the genomic coordinates the exons^38^.

Table 2 summarizes the relevant incidence data for de novo CNV from the three groups of children separately. We further partition CNVs into: ‘intergenic’, meaning not overlapping the transcript of any gene listed in RefGene; ‘coding’, meaning any event overlapping the coding regions of a RefGene; and ‘genic noncoding’, meaning any event that overlaps a transcript of a RefGene but without overlapping coding regions (Fig. 2).

**Table 2:** Role of de novo CNVs in simplex and multiplex autism.

		SSC unaffected		affected						
Effect	Group	CNV number	CNV rate	CNV number	CNV rate	Expected CNVs number	Delta	*p-*value	AD	PC
all	SSC affected	86	0.046	157	0.084	85.8	71.2	2 × 10^−06^	3.81% (2.16–5.47)	45.4% (29.2–57.9)
	AGRE affected			56	0.051	50.8	5.2	0.29	0.47% (−1.15–2.13)	9.3% (−29.3–34.4)
coding	SSC affected	44	0.023	106	0.057	43.9	62.1	4 × 10^−07^	3.32% (2.04–4.63)	58.6% (40.9–71.0)
	AGRE affected			34	0.031	26.0	8.0	0.12	0.72% (−0.52–2.06)	23.6% (−21.3–52.1)
intergenic	SSC affected	26	0.014	34	0.018	25.9	8.1	0.15	0.43% (−0.34–1.23)	23.7% (−24.9–55.6)
	AGRE affected			15	0.014	15.4	−0.4	0.52	−0.03% (−0.93–0.83)	−2.4% (−122.3–43.7)
genic noncoding	SSC affected	16	0.009	17	0.009	16.0	1.0	0.41	0.06% (−0.56–0.70)	6.1% (−92.9–57.3)
	AGRE affected			7	0.006	9.5	−2.5	0.74	−0.22% (−0.80–0.43)	−35.0% (−316.3–43.3)

The table presents our results for the contribution to autism of *de novo* CNVs as a whole (rows labeled ‘all’ in the “effect” column) and separately for the subsets of CNVs labeled as coding (CNVs that overlap with a coding exon), intergenic (CNVs that do not affect any genes) and genic noncoding (CNVs that affect genes but not coding regions). We analyzed only *de novo* CNVs of at least 4KB that we could detect with identical power in SSC and AGRE (see Supplementary Fig. 4). We used the number of children in a group as normalization factor to compute the “expected CNVs number” under the null model and we show the rate of the *de novo* CNVs in three groups in the “CNV rate” columns. Otherwise, we used the same three groups of children and the same statistics (delta, *p*-value, AD, PC) to quantify the role of a class of *de novo* mutation as described in Table 1’s legend.

In the SSC there is strong statistical evidence for an excess for events that disrupt coding regions (*p*-value = 4 × 10^−7^, AD = 3.32% (CI: 2.04 to 4.63), PC = 58.6% (40.9 to 71.0)). PC increases as copy number events hit more genes (see Supplementary Fig. 5), rising from 40% for events hitting one or more genes to 85% for events hitting five genes of more. The increasing impact of multigenic events has been previously noted^3^. There is no signal in the ‘intergenic’ or the ‘genic noncoding’ class (*p*-values = 0.15 and 0.41 respectively) for CNVs with size of at least 4 kb. In AGRE, there is no evidence for all CNV events (*p*-value: 0.29). There was a marginally significant excess of coding de novo CNVs (*p*-value: 0.12, AD = 0.72%, PC = 23.6%). This AD for coding CNVs in AGRE is about one quarter of the AD for SSC, with the difference being highly significant (*p*-value: 0.0007) using permutation testing.

#### Aggregated signals from de novo CNVs and LGDs

We analyzed the aggregated burden of de novo LGD and de novo coding CNVs in the affected of the SSC and AGRE by a permutation method (Table 3). The AD for the aggregated events is 9.80% (CI: 6.91 to 12.46, *p*-value = 5 × 10^−11^) for the SSC affected, and 2.15% (CI: −0.64 to 5.03, *p*-value = 0.062) for the AGRE affected. The aggregated ADs are roughly equal to the sum of the ADs for the individual event types. The aggregated AD for the affected children in the multiplex AGRE families is only marginally significant and its magnitude is a quarter of the AD for the simplex families in SSC. The direct comparison of the rates of the aggregated events shows that the two groups of affected children are significantly different (*p*-value: 10^−5^).

**Table 3:** Combined *de novo* LGDs and coding CNVs in simplex and multiplex autism.

Group	Events number	Expected events number	delta	*p*-value	AD
SSC affected	389	205.7	183.3	5 × 10^−11^	9.80% (6.91–12.46)
AGRE affected	150	126.2	23.8	0.062	2.15% (−0.64–5.03)

The table shows our joined analysis of the *de novo* LGDs and the coding *de novo* CNVs that allowed us to avoid the assumptions of independence between the two classes. The “events number” column shows to total number of *de novo* LGDs and *de novo* coding CNVs larger than 4KB in the two groups of affected children. The “expected events number” was computed by using the appropriate normalization factor for the two classes: number of *de novo* synonymous variants for *de novo* LGDs and the number of children for the coding *de novo* CNVs. Table 1’s legend describes the “delta”, “*p*-value”, and “AD” columns.

### Contribution of de novo intron mutations to children from simplex families

#### De novo CNVs

The ~30X WGS data enables us to observe smaller de novo CNVs than was possible with microarrays or WES. By simulation we show that, for the SSC, we have power of nearly 90% to detect events of size 1 kb from the WGS data, with equal power for both affected and unaffected children (Supplementary Fig. 4). In Table 4 we tabulate all single coding gene events further partitioned by class: coding and genic noncoding. The latter is divided into ‘intercoding intronic’, meaning exclusively between coding exons; and ‘peripheral’ meaning genic events overlapping UTRs or the introns splitting UTRs. There is clear signal from single gene events (*p*-value = 0.027), a finding that is predicted, but was not clear from our previous studies based on microarrays due to insufficient resolution. The AD 1.18% divides between coding (*p*-value = 0.12) and intercoding intronic (*p*-value = 0.0068). Although not statistically different, the PC for the intercoding intronic class (57.8%) is higher than for the coding class (24.6%). The peripheral regions show no signal, but the counts are low (5 in affected and 4 in unaffected). Other researchers report de novo targets in ‘regulatory’ domains in the periphery of genes^21^, and we discuss this later.

**Table 4:** Role of one-gene *de novo* CNVs in simplex autism.

	SSC unaffected		SSC affected						
effect	CNV number	CNV rate	CNV number	CNV rate	expected CNVs number	delta	*p*-value	AD	PC
all	53	0.028	75	0.040	52.9	22.1	0.027	1.18% (−0.03–2.34)	29.5% (−0.7–50.9)
coding	31	0.017	41	0.022	30.9	10.1	0.12	0.54% (−0.28–1.42)	24.6% (−16.4–53.7)
intercoding intronic	11	0.006	26	0.014	11.0	15.0	0.0068	0.80% (0.22–1.47)	57.8% (20.7–82.3)
peripheral	4	0.002	5	0.003	4.0	1.0	0.34	0.05% (−0.27–0.37)	20.2% (−304.9–100.0)

The increased power for CNV detection through whole-genome sequencing allowed us to examine the role small CNVs that affect a single gene. We analyzed all *de novo* CNVs including the ones smaller than 4KB and focused on the 1,874 unaffected and the 1,869 affected children from SSC. We quantified the role of all single-gene *de novo* CNVs (labeled as ‘all’) in the effect columns and separately for the non-overlapping subsets labeled as ‘coding’, ‘intercoding intronic’ and ‘peripheral’ (see Fig. 2 for definition of these terms). The format of the table and the analysis was identical to what is described in the Table 2’s legend.

#### Small de novo events

We identified extra support for the role of intercoding intronic regions by examining the de novo insertion-deletion events (indels) in intercoding intronic regions (IID) in SSC. These events were detected by the same sequence analysis pipeline we used to identify de novo LGD variants. The rate of de novo indels is influenced by age of parents (Supplementary Figure 6) and coverage, so we used de novo intergenic indels (IGID) as the normalization class for the comparison of incidences of de novo IIDs in the affected and unaffected children in SSC. We excluded the variants that directly affected the canonical splice-site regions. Our expectation of overall statistical signal was low, due to larger background and lower likelihood of impact for small events. Indeed, we see large numbers of intronic de novo indels in the SSC, with no signal of an ascertainment bias overall (Table 5, rows 1) in affected versus their unaffected siblings (*p*-values of 0.25).

**Table 5:** Role of *de novo* intercoding intronic indels (IID) and substitutions (ISB) in simplex autism.

			SSC unaffected		SSC affected						
	gene	event	normalization	functional	normalization	functional	expected functional				
gene set	number	type	number	number	number	number	number	delta	*p*-value	AD	PC
all genes	19,512	IID	5,859	3,916	5,768	3,932	3,855.2	76.8	0.25	4.11% (−7.52–15.85)	2.0% (−3.7–7.4)
autism LGD targets	748	IID	5,859	297	5,768	345	292.4	52.6	0.026	2.82% (−0.16–5.55)	15.3% (−1.0–28.0)
all NDD LGD targets	1,521	IID	5,859	560	5,768	645	551.3	93.7	0.008	5.01% (1.17–8.69)	14.5% (3.6–23.8)
autism missense targets	3,560	IID	5,859	1,153	5,768	1,171	1,135.1	35.9	0.25	1.92% (−3.38–7.33)	3.1% (−5.6–11.3)
autism synonymous targets	1,570	IID	5,859	561	5,768	569	552.3	16.7	0.30	0.89% (−2.89–4.90)	2.9% (−10.0–15.3)
all genes	19,512	ISB	58,274	37,814	59,340	38,057	38,505.7	−448.7	0.90	−24.01% (−59.04–15.00)	−1.2% (−2.9–0.7)
autism LGD targets	748	ISB	58,274	3,023	59,340	2,965	3,078.3	−113.3	0.92	−6.06% (−14.37–2.88)	−3.8% (−9.3–1.8)

We tabulated the numbers of *de novo* IID and ISB identified in the 1,874 unaffected and the 1,869 affected from the Simons Simplex Collection (the same groups as used in Table 4), separately for affected and unaffected children, and for five subsets of genes for the IID and two sets of genes for the ISB. We quantified the contributions to autism of each of the seven variants subsets. We used *de novo* intergenic indels (IGID) and *de novo* intergenic substitutions for normalization for the IID and ISB, respectively. The “functional number” columns show the number of IID or ISB and the “normalization number” columns show the number of IGID or IGSB. The methods and statistics presented (i.e. delta, *p*-value, AD, PC) are described in Table 1’s legend and in the Results section. Supplementary Data 5 shows the genes in each of the five sets. The first one is labeled “all genes” and represents all coding RefSeq genes. The other four are defined by the genes targeted by *de novo* mutations in children affected with one of these neuro developmental disorders (NDD): autism, schizophrenia, intellectual disability, developmental delay, or epilepsy. We used Denovo-db36 as a repository of the published *de novo* variants in these disorders. The “all autism LGD targets” refers to the genes targeted by *de novo* LGD mutation in a child with autism. The “all NDD LGD targets” are the genes with *de novo* LGDs in children with any of the five analyzed NDDs. The “autism missense targets” and “autism synonymous targets” are defined as genes with *de novo* missense and synonymous mutations respectively in children with autism.

To enhance our ability to detect signal, we therefore focused on the introns of genes that we expected to have a more favorable signal to noise ratio, namely those genes already identified as probable targets by prior studies. From Denovo-db^28^, we obtained two lists of genes targeted by de novo LGDs, either in children with autism (748 genes) or more generally in multiple neuro developmental disorders (1,521 genes) (see Table 5 and Supplementary Data 5). In both of these sets of genes, we see statistical signal for IID, with AD of 2.82% (CI: from −0.16 to −5.55) and *p*-value of 0.026 for the autism targets and AD of 5.01% (CI: from 1.17–8.69) and *p*-value of 0.008 for all neurodevelopmental disorder targets. In contrast, the control sets of genes show no significant difference of IID rates between affected and unaffected siblings. As with de novo CNVs, we see no signal from indels in the peripheral regions (See Fig. 2 and Supplementary Table 1).

We similarly examined the role of de novo intercoding intronic substitutions (ISB) using IGSB for normalization. We saw no signal for de novo ISB in either the overall intronic space or the introns of target genes. Given the enormous background of neutral substitutions, loss of signal from functional mutation is not surprising. We quantified our expectations by power calculations. The likelihood of observing statistically significant signal (*p*-value < = 0.05) from intronic substitutions in the intronic space is shown in Supplementary Figure 7, for all gene targets and for all candidate target genes. The power to detect a 5% contribution in a collection the size of the SSC is nearly 0 overall and around 0.2 for the autism target genes. Yet, it is unlikely that de novo intronic small indels contribute to the disorder while de novo intronic substitutions do not: the former cause greater damage per event, but the latter are about ten times more common.

We searched for mechanism of action by exploring the properties of the de novo INDs and ISBs that could reasonably be hypothesized to be contributory. These properties were: lengths of the indel; lengths of the affected introns; the proximity of the indel site to consensus splice sites; the degree of conservation at the mutated site; and the likelihood of creation of a new splice site. We also searched by the length of the largest open reading frame at the indel site, which might indicate the mutation affected an unannotated exon. We associated all de novo intronic events (both indels and substitutions) with each of the above properties, and then asked if the distributions of these properties differed significantly among subsets of the de novo events. These subsets were determined by mutation type (indel or substitution), the affected status of the child, and the target gene class (e.g., ‘all genes’ and ‘affected LGD targets’). None of our efforts produced a statistically significant signal. However, some of our observations were in a positive direction. Our entire analysis is reported in the Supplementary Note 2, Supplementary Table 2, and Supplementary Figure 8–34.

### Estimating the burden of de novo events to autism

Based on the results we have presented, we make a fresh estimate of the contribution of de novo events to autism incidence. We start with simplex families (Table 6). In this paper we estimate de novo CNVs contribute in ~4% of families. This is slightly lower than previous estimates, but our methods are very conservative. The estimates from LGDs (9%) and from missense (12%) derive from previous studies. These sum to 25%. In the present work we estimate the contribution from *de novo CNVs* and de novo small indels in the intronic space to be ~1% and ~5% respectively. Based on the study by An, et al.^21^ we further estimate contributory de novo promoter events at about 7%. The projected contributions from these two noncoding sources sum to 13%, about half the projected 25% contribution from coding events. Together they total 38%. We have not tallied certain de novo variant types, such as de novo intronic substitutions and structural variants at highly repetitive regions of the genome. Moreover, parental^29,30^ and somatic^31–33^ mosaicisms are often excluded in searches for de novo events. Thus, we may have underestimated contribution from de novo events. Given all the uncertainty, we estimate that de novo events of all types contribute to 35–45% of autism from simplex families.

**Table 6:** *De novo* contribution to autism in simplex families.

Type	Contribution
•**Coding**	25%
**∘CNV**	4%
**∘LGD**^**2**^	9%
**∘missense**^**2**^	12%
•**Non-coding**	13%
**∘Intronic**	6%
**·CNV**	1%
**small indels**	5%
**∘promoter**^**21**^	7%
**Total**	38%

The contributions to autism from simplex families of the various types of *de novo* coding and non-coding events are estimated from the literature or from this work. Contributions from *de novo* CNVs derive from this work. The contribution from small indels within introns is based on this work. The other contributions are estimates from the literature cited.

Next, we estimate the contribution of de novo variants separately for the high-risk and low-risk families. We can approximate the de novo contribution in the high-risk families, H, as equal to our estimate of the contribution in multiplex families. Based on our findings that the contribution in multiplex families is about a quarter of the contribution in simplex families (Table 3), and given our estimate of the contribution in simplex families, S = 35–45%, we estimate that H = 9–11%. The contribution to low-risk families, L, can then be computed using the estimates for the proportion of simplex families of low (pLs = 0.6) and high (pHs = 0.4) risk we made in Ronemus, et al.^15^. From the following equation:

4 $${{{{{\rm{S}}}}}}={{{{{\rm{pLs}}}}}}\;^*\; {{{{{\rm{L}}}}}}+{{{{{\rm{pHs}}}}}}\;^*\; {{{\rm{H}}}}.$$

we arrive at the estimate of the contribution to the low-risk families L = 52–67%.

Finally, using the de novo contributions in low (52–67%) and high-risk (9–11%) families and the estimate that half of autism cases originate from low-risk families and half from high-risk families^14,15^, we compute that the de novo contribution to overall autism is 0.5*(52–67%) + 0.5*(9–11%), or 30–39%. We arrive at a similar estimate using a second, more direct way for calculating the de novo contribution to autism overall that depends on the empirical measure of the proportion of autism cases in simplex and multiplex families. The growing SPARK collection provides such an estimate. In release 4 of the registered families there are 13,775 affected children from 6,505 multiplex families and 65,184 children from simplex families, giving us proportions of 0.17 (13,775 / (13,775 + 65,184)) cases in multiplex families and 0.83 cases in simplex cases. Combining these proportions with estimated de novo contributions in simplex and multiplex cases we arrive at 0.83 * 35–45% + 0.17 * 9–11% = 30.5–39.1%.

## Discussion

In our prior work^14,15^ we presented a simple model (the unified hypothesis) connecting de novo mutation and transmission in which we postulated high and low-risk autism families. The model explained the observation we made from analysis of multiplex families that the chance for autism is nearly 50% in a male born into a family with two or more prior children with autism^14^. This latter observation was confirmed by others using independent collections^13^, and is now confirmed in the latest release of the SPARK collection comprising more than 70,000 families with autism (work in progress). The model also explained our observation, based on a small study, that causal de novo mutation was relatively absent in multiplex families^1^. Unlike our observation of sibling risk in multiplex families, this result was contradicted in some other studies^5,7^. We felt these contradictory studies were flawed, having failed to take into account genetic drift of cells in culture. We resolve this question in the current study.

Unlike the samples of the SSC, in which DNA derives directly from blood, the DNA of many autism sample collections, including much of the AGRE, derive from cultured EBV infected lymphocytes. We demonstrated here that DNAs from some of the cultured cells of AGRE, though not all, display far greater cell-line genetic drift than seen in blood derived DNAs (see Fig. 1). Our results confirm the recent parallel study by Ruzzo et al.^18^ on the same data set. They too seek to remove artifacts of drift, although we use different methods. They filtered individual events, using machine learning techniques, while we filtered samples. The drift in samples creates the appearance of large numbers of de novo events in those samples, both substitutions and copy number, and we eliminated all events in such samples. When such samples are removed, the remaining samples from AGRE match the de novo variant characteristics for frequency and allele bias that we see in the SCC (Fig. 1).

With noisy samples removed, we observe, as did Ruzzo, et al.^18^, that the incidence of de novo LGDs in autism from multiplex families is significantly lower than the incidence in autism from simplex families (*p*-value: 0.001). In addition, we similarly observe a significant lower incidence for de novo CNVs that target genes (*p-*value: 0.0007). By combining these mutational types into one class, we then observed less contribution from de novo events in AGRE than in SSC affecteds, with a *p*-value of 10^−5^. These results therefore confirm our initial observations, based on the AGRE collection when it was still new, that causal de novo events are more prevalent in the simplex than in multiplex autism.

Yet we do see a higher incidence of de novo LGD and CNVs targeting genes in multiplex affected compared to the simplex unaffected. Neither alone has statistical difference. However, if we aggregate de novo CNVs and LGDs we find an elevated incidence with a *p*-value of 0.062. Here we differ from Ruzzo, et al.^18^ who examined only de novo LGDs in the same population and found no difference in incidence. Using the simplex unaffected as baseline, the ascertainment differential (AD) of de novo LGDs and CNVs targeting genes in AGRE are each about one quarter the AD for simplex affected.

WGS data enables us to directly examine the role of de novo variants in the parts of the genome that are non-coding. Although others have seen evidence of target in the promotor regions of genes using specialized search tools, in the absence of such tools we see no signal in the intergenic regions. However, our analysis does find evidence of a role for de novo CNVs in introns (*p*-value 0.0068, Table 4) and de novo small indels in introns of known neuro-developmental risk genes or the smaller subset of autism candidate genes (*p*-values 0.008, and 0.026, respectively, Table 5). We see no evidence for a role for de novo substitutions in this area, but this is expected from power considerations: there are vastly more substitutions than small indels or CNVs, and each of the substitutions would be of less impact than indels or CNVs (Supplementary Figure 7).

The ascertainment differential from CNVs is 0.80% and from small indels is 5.01%, yielding a combined AD of about 6% in simplex autism. Although, due to a limited power, we cannot observe a contribution from nucleotide substitutions in introns in the SSC population, we speculate that the contribution from substitutions within introns is of similar magnitude to that of the intronic small indels, or about 5%. Larger datasets would be needed to provide an empirically derived estimates from this source.

The extent to which introns were targets for disrupting de novo mutation, which we estimated at one half of the rate of disruptions in coding regions, surprised us. Given the complexities of splicing and gene regulation it is perhaps not a surprise that we saw no evidence for a predominant molecular mechanism. To understand these mutations better will require a case by case study of the RNA expression in the candidate cells, a study which is possible because almost the entire SSC cohort is also represented as immortalized cells.

We have made preliminary estimates of the contribution of de novo events to children with autism from low-risk (52–67%) and high-risk (9–11%) families, and to autism overall (30–39%). It is important to emphasize “preliminary” because, with the possible exception of de novo LGDs and CNVs in simplex collections, all the components used to make these estimates are not robust. Improved estimates would require many more families, more WGS on quads, and improved demographic data. However, 30–39% is possibly an underestimate, as it does not fully take into account mutations in highly repetitive regions, many other kinds of structural rearrangements, somatic mutations and parental mosaicism. Indeed, consideration of parental age and autism incidence would be more consistent with a substantially higher number (Taylor, et al.^34^ and studies in progress). Our estimates do not conflict with estimates of the proportion of families in which genetic transmission contributes. These latter estimates range variously from 40 to 90%^16,35–37^. As multiple mechanisms (de novo, transmission, epigenetic, and environmental) will contribute in many families, the sum of the proportionate contributions from each source over the entire population will exceed 100%.

## Materials and methods

### Whole genome datasets

The whole genome sequencing data for both the Simons Simplex Collection (SSC) and the Autism Genetic Resource Exchange (AGRE) were generated at the New York Genome Center. One lane of 150 paired-end reads on the Illumina 10X instrument was utilized per genome, achieving a depth of coverage of ~30X. We selected both affected and unaffected children from 1,878 of the SSC quad families (comprised of mother, father, an affected and an unaffected child) and 2,208 children (2,131 affected and 77 unaffected) from 859 nuclear families from AGRE for which we were able to identify both de novo small-scale and CNV variants. 279 of the AGRE children had DNAs extracted from whole-blood and the remainder had DNA extracted from LCLs. 1,869 affected and 1,874 unaffected children from the SSC and 1,107 affected and 36 unaffected children from AGRE passed the cell-line genetic drift filters. Supplementary Data 1 lists all children assessed in the manuscript. Since the study was limited to previously existing coded data, the CSHL IRB designated this study as “Not human subjects research” and therefore exempt from review.

### *De novo* substitutions and indels methods

We identified de novo substitutions and de novo short indels using the multinomial genotyper we have described previously^11^ and used extensively in our analysis of whole exome sequencing data from the Simons Simplex Collection^2,15,26^. To accommodate the lower coverage in the whole genome sequencing data, we set the filters for acceptable de novo candidate to denovoScr ≥70 and chi2Score > 0.001. The identified de novo variants in the cell-line genetic drift-free trios are listed in Supplementary Data 2 and Supplementary Data 3 for the SSC and AGRE, respectively.

### *De novo* CNV pipeline

We assembled a pipeline for the identifying de novo CNVs based on two different methods, “EWT de novo CNV finder” and “HMM de novo CNV finder,” both developed in house. Both methods analyze whole genome sequence data from a population of individuals jointly to deal with sample and regional biases, but are otherwise conceptually different. We combine the candidate de novo variants from the two methods and apply further stringent filters to guard against cryptic transmission. Detailed description of the de novo CNV pipeline components is presented in Supplementary Note 1, Supplementary Fig. 35, and Supplementary Data 6. We applied de novo CNV pipeline separately over the SSC and AGRE data and the final list of the de novo CNVs in the cell-line genetic drift-free children is presented Supplementary Data 4.

We compared our list of detected de novo CNVs to published de novo CNVs in the children we examined from SSC (Sanders, et al.^6^) and AGRE (Leppa, et al.^7^). We detected all 17 of the de novo CNVs reported in Leppa et al. and these are included in our analysis. We also detected all but 6 (likely due to differences in the sample labels) of the 204 de novo CNVs reported in Sanders et al. We also excluded 75 additional of the Sanders et al. de novo CNVs due to mosaicism detected in the parents, to polymorphisms in the parental population at levels above what our stringed filters allowed, or to children that did not meet our cell-line genetic drift filters. Supplementary Data 7 lists the Sanders et al. events not included in the current analysis and the reasons they were excluded. Supplementary Data 4, that lists the de novo CNV included in our analysis, indicates which of the de novo CNVs have been previously reported and clearly demonstrates that the WGS data enabled us to identify substantial number of new de novo CNVs smaller than resolution of the previous methods.

### Power to detect de novo CNVs

In our analysis, we compared rates for de novo CNVs across different groups children from the two collections, AGRE and SSC. Although the sequence data from the two collections had similar quality and coverage, it was essential to know if the small differences that existed affected the power to detect de novo CNVs. We used a simulation-based test for measuring the power to detect de novo CNVs from whole-genome data for a collection of trios.

The procedure used the bin values (the c_sb_ values described in Supplementary Note 1) generated from the “EWT de novo CNV finder” method and specifically tested the power to detect de novo deletions in male children using the EWT de novo CNV finder. We applied the test for deletions of 10 different sizes from 1 kb to 10 kb in three groups of children: the affected and unaffected males from SSC, and the affected males from AGRE. In each case, we simulated 10,000 autosomal deletions and checked how many of the simulated deletions could be detected by the EWT de novo CNV finder. Each simulated de novo deletion was generated using the following steps. First, we chose a random male trio; a random region of the given size on the X-chromosome that did not overlap with the pseudoautosomal regions, telomeric or centromeric regions; and a random region of the same size on one of the autosomes that did not overlap with telomeric and centromeric regions. We then took the real bin value matrix for the selected chromosome for all trios for the test groups of children (male and female), and replaced the bin values for the male child of the selected trio with the bin values from the selected X-chromosome region for the same child. The updated matrix was fed to the EWT de novo CNV finder. Finally, for each simulated deletion we checked if the selected autosomal region overlapped with any of the candidate de novo deletions for the selected child generated by the finder. See Supplementary Figure 4 for results.

### Power to detect de novo substitutions given different coverages

We measured the power to detect de novo substitutions separately for each trio from the SSC and AGRE collection (Supplementary Fig. 2 and Supplementary Data 1). For every trio we selected 10,000 random genomic positions and obtained the number of reads covering each of these positions in the mother, father, and the child (coverage). We then simulated a count data for a de novo mutation. Parents had counts for the reference allele equal to the observed coverage and 0 reads supporting the alternative allele. We used a random value from a binomial distribution Binomial (*p* = 0.47, *N* = coverage for the child) to obtain a count for the alternative allele for the child. The count for the reference allele was that set to the difference of the child’s coverage and the count for the alternative allele. The resulting 2 (reference and alternative allele) by 3 (mother, father, child) count matrix was fed to the multinomial genotyper. The proportion of the 10,000 positions successfully classified as a de novo variant by the multinomial genotyper was used as an estimate of the power to detect SNV in for the trio.

### Reporting summary

Further information on research design is available in the Nature Research Reporting Summary linked to this article.

## Supplementary information

Supplementary Information.

Description of Additional Supplementary Files

Supplementary Data 1

Supplementary Data 2

Supplementary Data 3

Supplementary Data 4

Supplementary Data 5

Supplementary Data 6

Supplementary Data 7

Reporting Summary

## Data Availability

We obtained existing whole-genome sequence datasets for the Simons Simplex Collection (SSC) and the AGRE collections that have already been used in published manuscripts^18,21,25^. The SSC whole-genome sequence data can be obtained from Simons Foundation Research Initiative (SFARI; https://www.sfari.org). The AGRE whole-genome sequence data can be obtained from the Hartwell Foundation’s Autism Research and Technology Initiative (iHART; http://www.ihart.org). Access to these resources is subject to approval by the respective institutions. We used these whole-genome sequencing data to identify de novo substitutions, de novo small indels, and de novo CNV using the methods described above and in Supplementary Note 1. We provide the complete lists of children and identified de novo variants together with all attributes used in our analysis in Supplementary Data 1–5.

## Code availability

The source code we used in our analysis is included in the public GitHub repository https://github.com/iossifovlab/denovoInHighAndLowRiskPaper. In addition, we provide the input data for these scripts together with the results of executing the scripts over the provided input. The input is a copy of the Supplementary Data associated with this manuscript, and we include it in the source code repository for convenience. Thus, interested readers can: (1) study the code and the complete set of results that we discuss in the manuscript, (2) re-run the scripts on the provided input to reproduce precisely our analysis, and (3) use the scripts over input related to their work to perform similar analyses. The official release (v1.0) of the repository can be accessed through 10.5281/zenodo.5137986.
