# Supplementary material for: Rates of contributory de novo mutation in high and low-risk autism families
Source: Commun Biol. 2021 Sep 1;4:1026. doi: 10.1038/s42003-021-02533-z (PMC8410909; doi:10.1038/s42003-021-02533-z)
Supplement: Supplementary file 1 — Supplementary Information. [file 42003_2021_2533_MOESM1_ESM.pdf]

Supplementary Information

Rates of contributory *de novo* mutation in high and low risk autism families

Seungtai Yoon[1,*], Adriana Munoz[1,*], Boris Yamrom[1], Yoon-ha Lee[1], Peter Andrews[1], Steven Marks[1], Zihua Wang[1], Catherine Reeves[2], Lara Winterkorn[2], Abba M. Krieger[3], Andreas Buja[3], Kith Pradhan[4], Michael Ronemus[1], Kristin K. Baldwin[5,6], Dan Levy[1], Michael Wigler[1,2], and Ivan Iossifov[1,2,+]

[1]Cold Spring Harbor Laboratory, Cold Spring Harbor, NY, USA

[2]New York Genome Center, New York, NY, USA

[3]Statistics Department, The Wharton School, University of Pennsylvania, Philadelphia, PA, USA

[4]Department of Medicine, Albert Einstein College of Medicine, Montefiore Medical Center, Bronx, NY, USA

[5]Department of Neuroscience, The Scripps Research Institute, La Jolla, CA, USA

[6]Department of Genetics and Development, Columbia University, New York, NY, USA

* equal contributions

+ corresponding author: iossifov@cshl.edu

Table of Contents

Supplementary Note 1. *De novo* CNV pipeline 4
EWT *de novo* CNV finder 4
Definition of the bin and region scores 4
HMM *de novo* CNV finder 5
Merging of fragmented CNV calls 6
Additional population filter 6
Supplementary Note 2. Functional Analysis of *de novo* intronic events 7
Properties 7
Variant size 7
Intron length and distance to the nearest splice site 7
Open Reading Frame length 8
Conservation scores 8
Simple splice site scores 8
Scores based on the machine learning splice site prediction tool SpliceAI 9
Supplementary Figures 10
Supplementary Figure 1. *De novo* CNVs in families with and without cell-line genetic drift 10
Supplementary Figure 2. Power for detection of *de novo* substitutions 11
Supplementary Figure 3. Parental ages 12
Supplementary Figure 4. Power for detection of *de novo* deletions 13
Supplementary Figure 5. Percent Contributory (PC) by CNV gene number 14
Supplementary Figure 6. Rates of *de novo* substitutions and indels vs age 15
Supplementary Figure 7. Power for detecting contribution from intronic substitutions 16
Supplementary Figure 8. Variant size distributions 17
Supplementary Figure 9. Intron length distributions 18
Supplementary Figure 10. Distance from splice site distributions 18
Supplementary Figure 11. ORF length distributions 19
Supplementary Figure 12. SpliceAI DS_AG score distributions 19
Supplementary Figure 13. SpliceAI DS_AL score distributions 20
Supplementary Figure 14. SpliceAI DS_DG score distributions 20
Supplementary Figure 15. SpliceAI DS_DL score distributions 21
Supplementary Figure 16. SpliceAI MAX_DS score distributions 21
Supplementary Figure 17. Acceptor alt score distributions 22

Supplementary Figure 18. Acceptor ref score distributions 22
Supplementary Figure 19. Acceptor alt-ref score distributions 23
Supplementary Figure 20. Donor alt score distributions 23
Supplementary Figure 21. Donor ref score distributions 24
Supplementary Figure 22. Donor alt-ref score distributions 24
Supplementary Figure 23. phylop, 100 vertebrates score distributions 25
Supplementary Figure 24. phylop, 30 vertebrates score distributions 25
Supplementary Figure 25. phylop, 20 vertebrates score distributions 26
Supplementary Figure 26. phylop, 7 vertebrates score distributions 26
Supplementary Figure 27. phastCons, 100 vertebrates score distributions 27
Supplementary Figure 28. phastCons, 30 vertebrates score distributions 27
Supplementary Figure 29. phastCons, 20 vertebrates score distributions 28
Supplementary Figure 30. phastCons, 7 vertebrates score distributions 28
Supplementary Figure 31. CADD score distributions 29
Supplementary Figure 32. Minimum property rank distributions 29
Supplementary Figure 33. Splice-site model 30
Supplementary Figure 34. An example of acceptor splice-site sequence score 31
Supplementary Figure 35. HMM *de novo* CNV finder 32
Supplementary Tables 34
Supplementary Table 1. Indels and Substitutions in Peripheral Regions 34
Supplementary Table 2. Results of the functional analysis 35
Supplementary References 37

Supplementary Note 1. *De novo* CNV pipeline

We used two different methods developed in house, “EWT *de novo* CNV finder” and “HMM *de novo* CNV finder,” to identify *de novo* copy number variation candidates. We then merged the candidate *de novo* events from the two methods and applied a series of stringent population filters to guard against cryptic transmission. These steps are described in detail below.

EWT *de novo* CNV finder

We obtained the depth of coverage at each genomic position for each sample. We split the genome in 100bp bins and for every bin and every sample we assigned a bin value (c_{sb}) equal to the sum of the coverage depths for the positions in the bin (see “Definition of the bin and region scores” below). Bins with average values across the population (c_b) of less than 400 (or average depth of less than 4) were removed from the analysis. We divided each bin value by the median across the bins from the same chromosome and sample (n_{sb}), and we calculated z-scores (z_{sb}) for each bin throughout the population. We applied event-wise testing (EWT) for segmentation to the z-scores for each chromosome from each individual[1]. The result is a list of gain and loss regions for each sample. One child from the SSC and 11 from AGRE with too many gains and losses were removed. Each region is characterized by the first and last bin, by the average of the z-scores for the sample bins (z_{sr}), and by the “region population z score,” computed as the z score of the region’s z-scores for all samples (zz_{sr}). We tested each region from every child to determine whether it was a *de novo* copy number event using the following criteria: 1) We filtered out candidates that were <20 bins (2 kb) long and had the median bin values (c_{sr}) of <200 to avoid homozygous deletions. 2) Variants were called as *de novo* when the absolute value of the region population z score (zz_{sr}) of a child was greater than 6 and those of parents were less than 1.5. Children with *de novo* events in 8 (for the SSC) and 5 (for AGRE) or more chromosomes were filtered out. 3) By visual inspection of the plots, we flagged obvious false positives that could not be caught in the previous filtering steps such as CNVs of cryptic transmission. 1614 out of 2076 and 1366 out of 1780 were removed by visual inspection for the SSC and AGRE, respectively.

Definition of the bin and region scores

Bin (b) properties

c_{sb}	*bin value* for sample s and bin b:
c_b= mean(c_{sb} for all s)	*average bin value* for bin b:
$t_{sb} = c_{sb}$ / median($c_{sb'}$ for chrom(b’)=chrom(b))	*bin ratio* for sample s and bin b:
z_{sb} = [t_{sb} – mean(t_{sb} for all s)] / std(t_{sb} for all s)	*bin z-score* for sample s and bin b:

Region (r) properties

z_{sr} = mean(z_{sb} for b in r)	*region z-score*
zz_{sr} = [z_{sr} – mean(z_{sr} for all s)] / std(z_{sr} for all s)	*region population z-score*
c_{sr} = median(c_{sb} for b in r)	*median bin value*
t_{sr} = median(t_{sb} for b in r)	*region median ratio*
p_r = median(t_{sr} for all s)	*ploidy measure*

HMM *de novo* CNV finder

We first partition the genome into variable-sized bins with similar expected number of reads starts. For each base pair in the human genome, we count the number of read starts per individual and then calculate the median over the population. Then, starting from the beginning of each chromosome, we advanced a base pair at a time until the sum of the median number in the interval exceeds 20. We recorded the end of the current bin and start the next interval at the following base pair. Following this procedure, the genome was split into about 9 million bins with a median length of 300 bp per bin. We then counted the read starts within the bins for each individual and stored the results into a count matrix ***C***.

We developed and ran the "HMM/SVD EM algorithm" separately for each chromosome (Supplementary Figure 35). The algorithm is based on a Hidden Markov model (HMM)[2] with length equal to the number of bins within the chromosome. The hidden states were allowed to be one of S = 0, 1, 2, 3, or 4, representing the number of copies the person had at each bin. The transition probabilities are based on a global transition matrix, ***T***, representing the transition probabilities between states of neighboring genomic positions. The matrix ***T*** is parametrized by 5 parameters (a, b, c, d and e), and its structure and the definitions of the parameters are shown in Supplementary Figure 35, panel A. The transition probabilities between bins are computed using the distance (in base pairs) between the middle positions of neighboring bins. The initial probability for the HMM is set to the equilibrium state distribution induced by ***T***. The emission probabilities for the HMM are modeled as a Poisson distribution $E(c_{pb}|S) \propto Poisson(\lambda_{pb}S^{*})$, where λ_{pb} is per-person and bin-specific emission rate parameters, and S* = max(0.1, S). We combine all of λ_{pb} into a matrix ***L***.

The "HMM/SVD EM algorithm" estimates the emission rate parameters, ***L***, (the expected numbers of read-starts in a bin in a person per one copy of the related genomic region) and the bin-genotype matrix, ***H***, representing the copy number state for each person and bin. We begin by assuming a universal copy number state of 2 by creating a constant bin-genotype matrix ***H*** (which equals 2 for all p and b). We then calculate the emission rates (***L***) by dividing the counts, ***C***, by the bin genotypes (***H***). To avoid over-fitting the emission rates, we use a low-resolution approximation based on the 3 components with maximal eigenvalues in the SVD normalization (Supplementary Figure 35, panel B). The normalization accounts for sample batch effects, regional GC content and other systematic effects. Unfortunately, common copy number polymorphisms also affect the emission rate estimations. To remedy this, we ran a Viterbi algorithm on the HMM defined by the normalized emission rates and the observed counts to identify the most likely copy state path through every bin for each person (***H***). We then repeat the process using the updated copy number states ***H***. This ensures that in the next step, the rate estimates will be distorted less by existing copy number polymorphism. The details of the iterative procedure are formally defined in Supplementary Figure 35, panel C. Finally, the HMM/SVD EM algorithm outputs the latest rate matrix **L** and bin-genotype matrix **H**.

We then analyzed the bin-genotype of every trio (mother, father, and child) to identify candidate *de novo* CNV events. This was done in two steps. First, we represented the trio bin-genotype into a simpler form where states 0 and 1 are represented as -1 (deletion), the ground state 2 is represented as 0 (or expected copy number state), and states 3 and 4 are represented as 1 (duplication). Second, in the simplified genotype representation, we looked for consecutive bins that all had the same non-zero state for the child (either 1 or -1) and the 0 genotype for the parents (Supplementary Figure 35, panel D). All

such series of bins, represented as the first and last bins and the trio, are listed as candidate *de novo* duplications (if the child's simplified genotype was 1) or deletions (if the child's genotype was -1).

Finally, we subjected the candidate *de novo* events to a series of filters to decrease the false positive rates. (1) We removed all candidates that contain less than 20 bins. (2) We summed the number of reads starts in the bins of the candidate *de novo* CNV for each of the three family members, and removed candidates if a family member has less than 2 reads within the events. (3) For each of the candidate event bins, we computed the number of parents in the analyzed population that have bin-genotype different than 2 (bin out-of-ground parents). We remove the candidate, if the mean out-of-ground parents, across the candidate bins is larger than 20. (4) To avoid false positive *de novo* CNV calls due to cryptic transmission, to mosaic parents, or to artifact-prone genomic regions, we imposed a requirement for the observed counts to closely match the expected counts based on the HMM models for the members of the trio. We define the *deviation from copy 2* as $\left|\frac{\sum_{b \text{ in the candidate region}} c_{pb}}{2 * \sum_{b \text{ in the candidate region}} \lambda_{pb}} - 1\right|$, for a person p and the region of the candidate *de novo*. We then required that the deviation is less than 0.075 in both parents and larger than 0.425 for the child.

Merging of fragmented CNV calls

For each individual, we collected all the CNV calls derived from two methods. Then we grouped events that are less than 5 Mb apart and applied unique identifiers ("merge index") dealing with deletions and duplications separately. Given low rates of *de novo* CNVs, it is highly unlikely to have independent *de novo* events in the same child within 5 Mb. The more likely explanation is that such candidates represent the same underlying event (Supplementary Data 6).

Additional population filter

As an additional guard against potential transmission, we applied two additional strong population filters. Both filters were based on the scores computed by the "EWT *de novo* CNV finder." First, we removed any deletion event seen six or more times as a deletion event in the parent population, and similarly for duplications. A candidate *de novo* CNV was considered present in a parent if the "region median ratio" (t_{sr}), defined as a median of the bin ratios (t_{sb}) for the bins in that parent and that region, is less than 0.7 for the deletions or greater than 1.3 for duplications.

Second, we kept only those events (marked as "diploid") where the great majority of parental genomes have a diploid copy number count. The "ploidy measure" for a region (p_r) is the median of region median ratios (t_{sr}) for the same region across the whole population. We consider regions with ploidy measure between 0.85 and 1.15 to be mostly diploid in the population.

Supplementary Data 6 shows the number of parents and the ploidy measure for all de novo CNV candidates.

Supplementary Note 2. Functional Analysis of *de novo* intronic events

We observed that in the affected children from the SSC there were significantly more *de novo* intronic indels in the autism target genes than were found in unaffected siblings. We inferred that this increase is due to the indirect ascertainment of intronic indels that contributed to diagnosis of autism in the affected children, and we asked whether contributory *de novo* intronic indels could be distinguished from the non-contributory events by some of their properties. We examined 24 numerical properties (see the detailed list and description below) that could reasonably be hypothesized to point to contributory events. We associated all *de novo* intronic events (both indels and substitutions) with each of the 24 properties. We also computed "min-rank" score for each *de novo* event that combined the 24 raw scores separately for *de novo* inter-coding intronic indels (IID) and *de novo* inter-coding intronic substitutions (ISB). All IIDs were ranked (ordered) independently by each of the 24 properties such that the indel with the most damaging property value was assigned rank 1. Every IID was then assigned the minimum rank it achieved across the 24 score ranks. The min-rank score for the ISBs was computed in the same way.

We tested if the distributions of the 24 properties and the min-rank scores differed among subsets of the *de novo* events defined by the *de novo* intronic event type (indel or substitution), the affected status of the child carrying the *de novo* events (affected or unaffected), and by the class of the gene targeted by the event ("all genes" or "autism target genes").

For each property, we performed four tests, two related to IIDs and two related to ISBs. The resulting p-values are shown in Supplementary Table 2. First, we compared the property values for the IIDs that fall into neurodevelopmental candidate genes identified in the affected children to the property values of the IIDs in the same set of genes in the unaffected children. We used Mann-Whitney test[3] to compare the two set of values and presented the resulting p-values for each property in the "IID / target genes" column of Supplementary Table 2. Second, using the same approach, we compared the distributions of the property for all IIDs from affected children to that in unaffected children ("IID / all genes" column). Similarly, we compared the distributions of the property for *de novo* intronic substitutions from the affected and the unaffected children in the neurodevelopmental candidate genes ("ISB / target genes") and in all genes ("ISB / all genes").

A more detailed view of the distributions of each of the properties over the various classes of events can be seen in Supplementary Figures 8-32.

Properties

Variant size

For every *de novo* intronic variant, we computed the genomic span of the variant in base pairs (Supplementary Figure 8). We assign size of 0 for the *de novo* substitutions. For the *de novo* deletions and insertions, we assign a size equal to the number of deleted or inserted bases, respectively.

Intron length and distance to the nearest splice site

For every *de novo* intronic variant, we identified the shortest intron covering the variant. We recorded the length of the shortest intron ("intron length" property; see Supplementary Figure 9). We also recorded the distance between *de novo* events and the splice sites of the shortest intron that was closest to the observed event ("distance from splice site" property). If the closer splice site was the donor site, we assigned positive numbers, and we assigned negative numbers if the closer splice site was

the acceptor site. We tested if the absolute value of the distance from splice site was different between the various classes of the *de novo* mutations (Supplementary Figure 10).

Open Reading Frame length

To test if the *de novo* intronic events fall in and disrupted cryptic coding exons, we looked for a bias in the size of the largest open reading frame in the direction of transcription (see "ORF length" property) among the difference lasses of *de novo* events (Supplementary Figure 11).

Conservation scores

We used two methods for measuring conservation: phastCons[4] and phyloP[5]. The two methods compute a conservation score for each genomic location based on a given phylogenetic three. We downloaded the computed scores from the two methods over four different phylogenetic trees based on 100, 30, 20, and 7 species from the UCSC genome browser. (Supplementary Figures 23-30).

In addition, we used CADD scores. We downloaded the computed scores from the CADD website[6] (Supplementary Figure 31)

Simple splice site scores

To test if the *de novo* intronic mutations created novel splice sites, we developed donor and acceptor splice site sequence scores for short sequences (see below for a detailed definition of the scores). We computed these two scores for the reference sequence 5 base pairs upstream and downstream of a location where a *de novo* event occurred ("ref" scores), and separately for the local sequence after the *de novo* event was introduced ("alt" scores). We also computed the differences between the alt and ref scores. Thus, every *de novo* intronic mutation was associated with six splice-site sequence scores: "ref," "alt" and "alt-ref" for both donor and acceptor splice-site scores (Supplementary Data 2 and 3). We tested each of the six scores for their ability to discern *de novo* intronic events in affected children in target genes (Supplementary Table 2 and Supplementary Figures 17-22).

Definition of the donor and acceptor splice-site sequence scores

We defined position-specific sequence models for donor and acceptor splice sites based on a 20-bp sequence context (10 bp upstream and 10 bp downstream of the splice site). We measured the frequency of the four nucleotides at each of the 20 positions independently using the ~200,000 annotated donor and acceptor sites in the RefSeq[7] database: $f_{pn}^{\mathcal{D}}$and $f_{pn}^{\mathcal{A}}$, where $\mathcal{D}$ is for donor, $\mathcal{A}$ is for acceptor, p is index for the position and n is A, C, G or T. We also measured the frequency of the random intronic nucleotides, $f_n^{\mathcal{R}}$, and defined the position specific donor and acceptor splice-site scores as log-likelihood ratios:

$$\text{DS(context)} = \log\frac{L(\text{context}|\mathcal{D})}{L(\text{context}|\mathcal{R})} = \sum_{p=1}^{20} w_{pn_p}^{\mathcal{D}} \text{ and}$$

$$\text{AS(context)} = \log\frac{L(\text{context}|\mathcal{A})}{L(\text{context}|\mathcal{R})} = \sum_{p=1}^{20} w_{pn_p}^{\mathcal{A}},$$

where "context" is the 20 bp sequence context around a candidate splice site position, L(context|M) is the likelihood function for the context given a specified model M under the assumption of independence among the context positions, $n_p$is the p-th nucleotide in context, $w_{pn}^{\mathcal{D}} = \log\frac{f_{pn}^{\mathcal{D}}}{f_n^{\mathcal{R}}}$, and $w_{pn}^{\mathcal{A}} = \log\frac{f_{pn}^{\mathcal{A}}}{f_n^{\mathcal{R}}}$ (Supplementary Figure 33).

Finally, we defined the donor and acceptor splice-site sequence scores for a given short sequence, “seq,” as the maximum of the position-specific splice-site scores over all positions in seq:

DS(seq) = max DS(context) for context in seq;

AS(seq) = max AS(context) for context in seq.

See Supplementary Figure 34 for an example of the AS scores for the “ref” and “alt for a *de novo* intronic insertion.

Scores based on the machine learning splice site prediction tool SpliceAI

In addition to the simple splice site model presented above that uses only local 20bp long context, we used the machine-learning based splice site prediction tool SpliceAI[8]. SpliceAI uses much larger sequence context of 10Kb and has been shown to have superior ability to predict splice sites locations and gains and losses of splice sites caused by genetic variants compared the alternative approaches. Using SpliceAI, we associated each of the genic *de novo* indels and substitutions we identified with five scores:

DS_AL – probability for a loss of an acceptor site;

DS_AG – probability for a gain of an acceptor site;

DS_DL – probability for a loss of a donor site;

DS_DG – probability for a gain of a donor site;

MAX_DS – the maximum of the DS_AL, DS_AG, DS_DL, and DS_DG probabilities.

Supplementary Figures 12-16 show the distributions of these properties and the results of the comparisons of these properties for variants in affected and unaffected children.

Supplementary Figures

Supplementary Figure 1. *De novo* CNVs in families with and without cell-line genetic drift

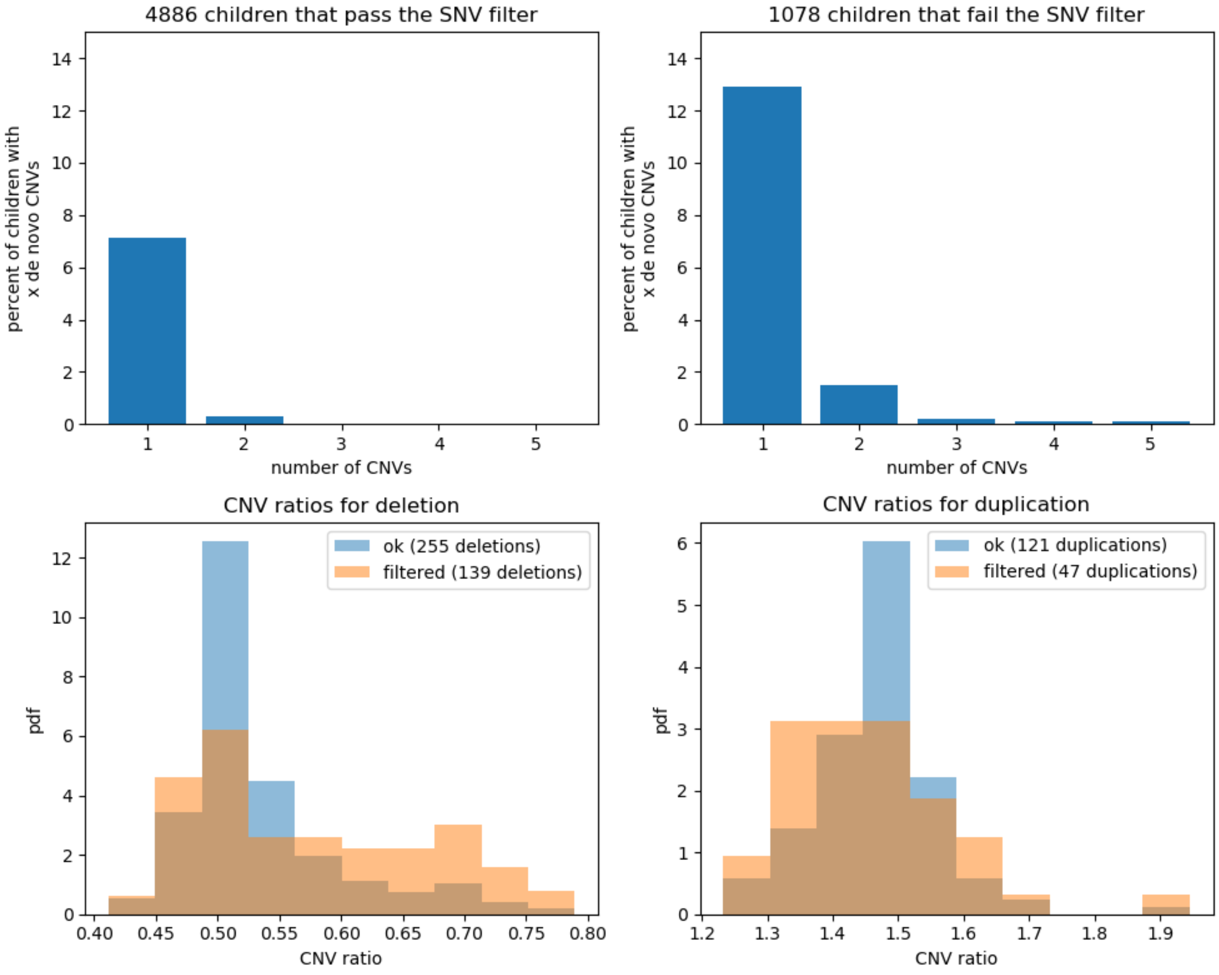

The top panels show the normalized histograms of the numbers of children with given numbers of *de novo* CNVs for the affected and unaffected children from SSC and AGRE. At top left are those that we consider free of cell-line genetic drift; at top right are the children that exhibit cell-line genetic drift artifacts. See Figure 1 for further details of how we identify the cell-line genetic drift-free children. Children free of cell-line genetic drift have a much lower rate of *de novo* CNVs.

The bottom panels show distributions (pdf) of the region median ratios (t_{sr}; see the Supplementary Note 1 for formal definition) of deletions (bottom left) and duplications (bottom right). Pure (non-clonal) deletions and duplications are expected to have region median ratios of 0.5 and 1.5, respectively. Blue and orange bars are for CNVs identified in children with ("ok") and without ("filtered") detected cell-line genetic drift, respectively.

Supplementary Figure 2. Power for detection of *de novo* substitutions

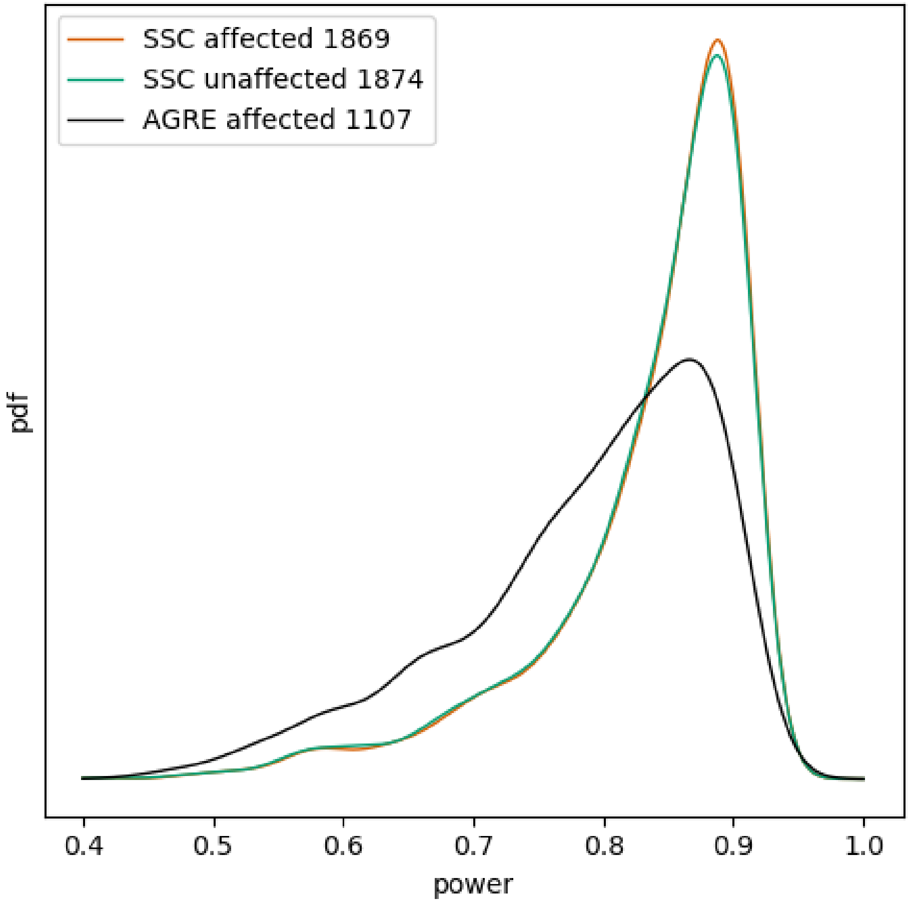

We computed the power to detect *de novo* substitutions separately for each of the SSC and AGRE children through simulation (see Materials and Methods). The power depends closely on the coverage and sample preparation protocols. The figure shows the distribution of the powers for three subsets of children that we considered free from cell-line genetic drift: the affected (orange) and unaffected (green) children from SSC and the affected children from AGRE (black). While affected and unaffected children from the SSC have similar distribution of powers, the affected children from AGRE have a distribution enriched for children with lower power.

Supplementary Figure 3. Parental ages

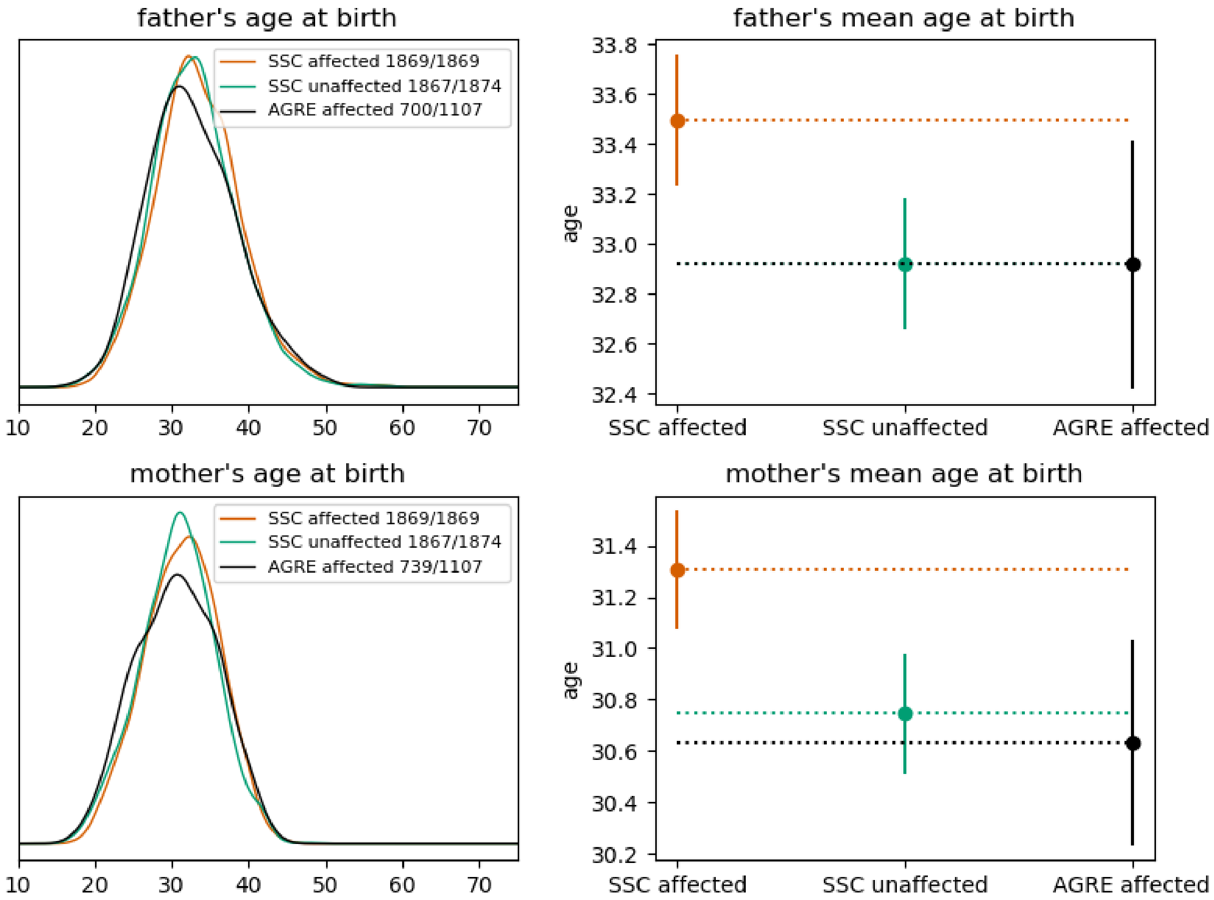

The top left panel shows the distribution of the ages of the father (when available) at the birth of the child for three groups of children: the affected (orange) and unaffected (green) children from the SSC and the affected children from AGRE (black). The legend within the panel shows the number of children in each group (left) and the number of these for which we know the ages of the father (right). The top right panel shows the mean ages for each of the three groups, together with a 95% confidence interval for the mean. The bottom left and right panels show the same information for mothers of the same three cohorts.

Supplementary Figure 4. Power for detection of *de novo* deletions

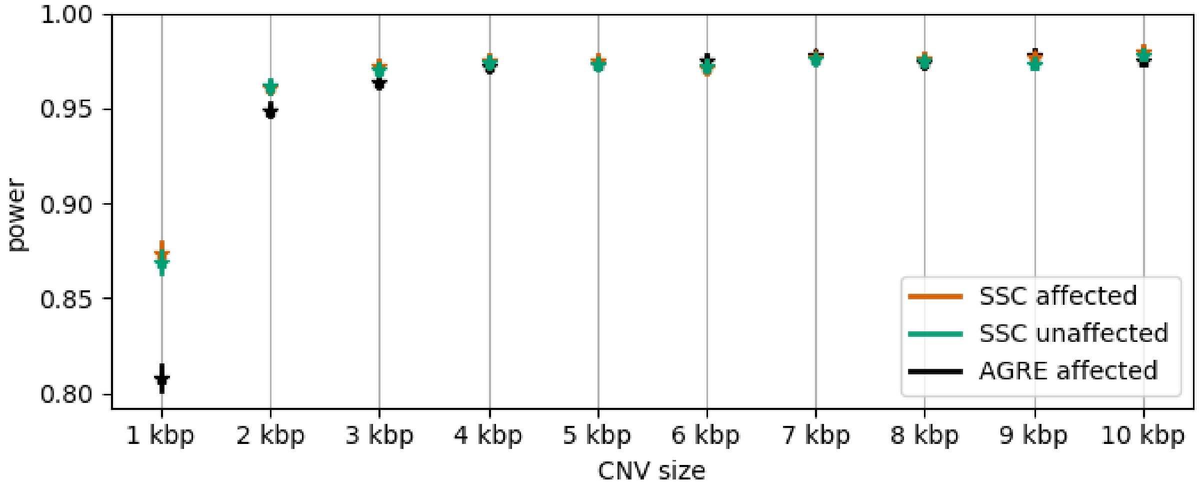

We show measurements of the power to detect *de novo* deletions (Y-axis) of various sizes (X-axis) for three groups of children: the affected (orange) and unaffected (green) children from SSC, and the affected children from AGRE (black). Also plotted are 95% confidence intervals for all measurements. The simulation-based method for measuring the power for deletion detection is described in the Materials and Methods. The power is virtually identical between affected and unaffected children from the SSC across all sizes. The power for AGRE children is the same as for the SSC children for larger deletions (≥4 kb), but it is significantly lower for smaller deletions.

Supplementary Figure 5. Percent Contributory (PC) by CNV gene number

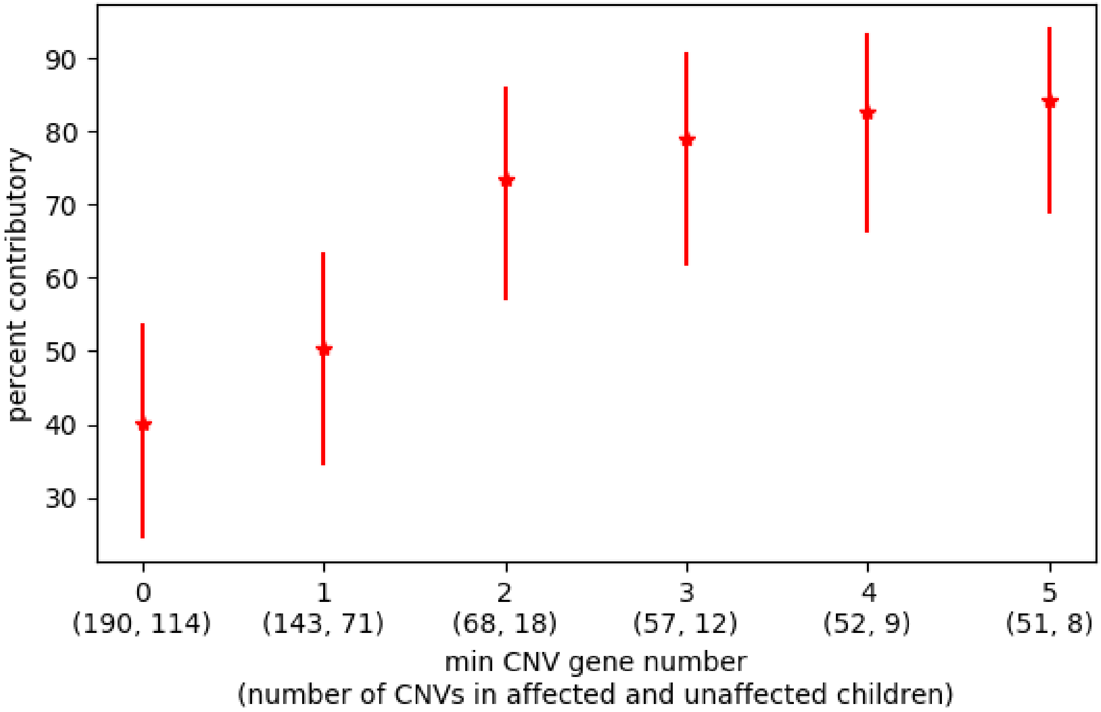

The "percent contributory" measure is our estimate of the percent of the *de novo* events of a particular class identified in the affected children that have contributed to the disorder. The procedure to compute the percent contributory is described in the Results section, and it is based on comparing the rates for the *de novo* events in affected and unaffected groups of children. The figure shows the percent contributory estimates (plus their 95% confidence interval) in the affected children from the SSC of all *de novo* CNVs (min CNV gene number = 0), CNVs affecting one or more genes (min CNV gene number = 1), CNVs affecting two or more genes (min CNV gene number = 1), and so on. The X-axis is annotated with the number of *de novo* CNVs of the particular class observed in 1,869 affected and 1,874 unaffected children from the SSC.

Supplementary Figure 6. Rates of *de novo* substitutions and indels vs age

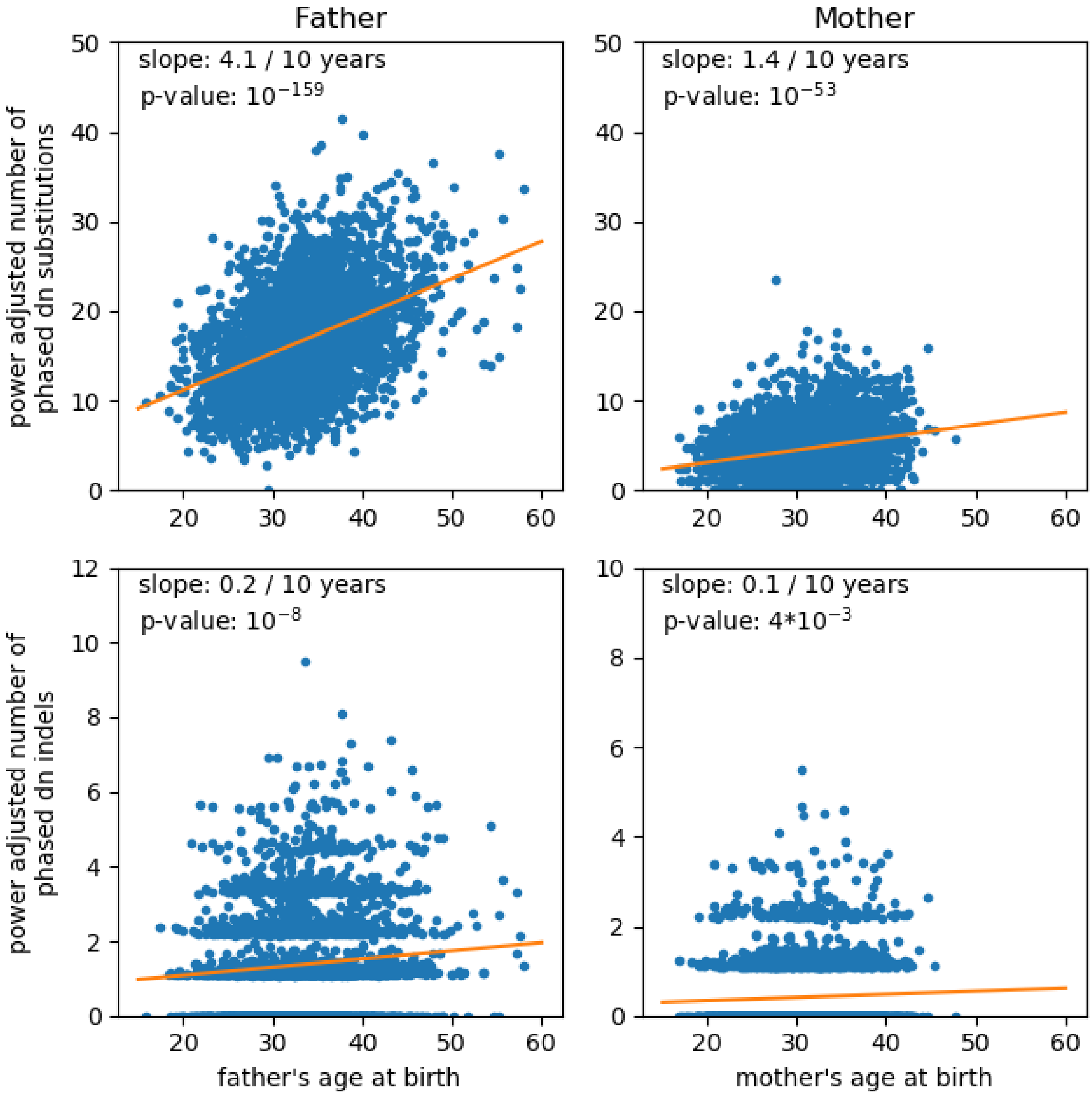

We plotted the power-adjusted numbers (on the Y-axis) of *de novo* SNVs (top panels) and indels (bottom panels) in all affected and unaffected children from the SSC that we determined to originate from the paternal (left panels) or maternal (right panels) haplotypes. The power adjustment is described in the legend of Figure 1. On the X-axis, we plotted the age of the fathers and the mothers at the birth of the child. We fit a linear regression for the adjusted number of *de novo* events and ages for each of the four plots, and we show the resulting slope and the p-value under the null model of a 0 slope. As has been reproduced numerous times, it is clear that the number of *de novo* SNVs increases with the age of both the father and the mother[9-12]. The dependencies between the number of *de novo* indels and the age of the father and the age of the mother are also significant.

Supplementary Figure 7. Power for detecting contribution from intronic substitutions

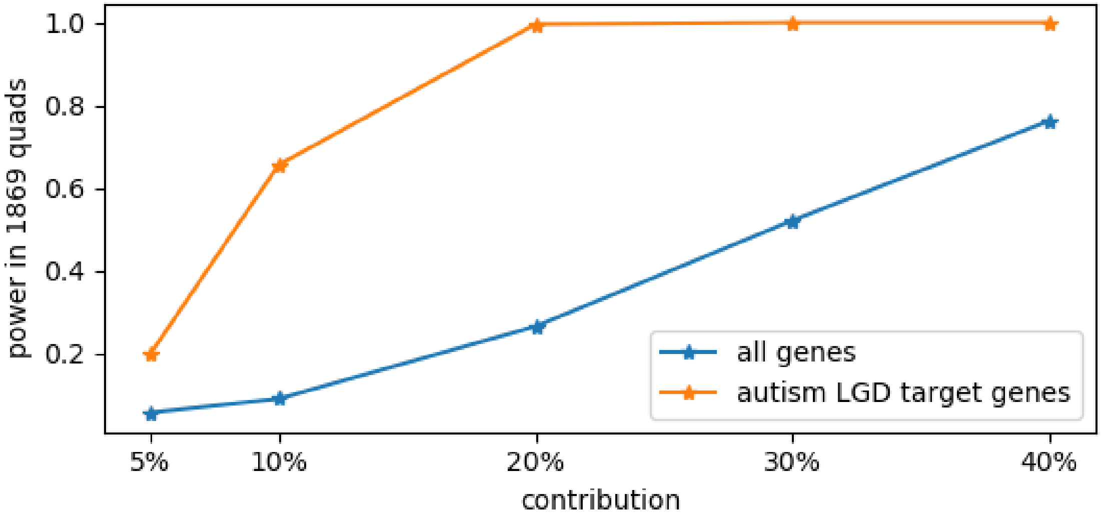

We show the power to detect contribution from *de novo* intronic substitutions in a simplex collection with 1,869 affected children (the number of affected children from SSC we analyze in this manuscript) under several hypothetical levels of contribution from intronic substitutions in “all genes” (blue curve) or in the “autism LGD target genes” (orange curve). In all cases the power is measured using 0.05 significance. The background rates of *de novo* events per child measured in the unaffected children from the SSC determine these curves. For all genes that background rate is 20.1, and for autism LGD target genes that rate is 1.6 per child.

Supplementary Figure 8. Variant size distributions

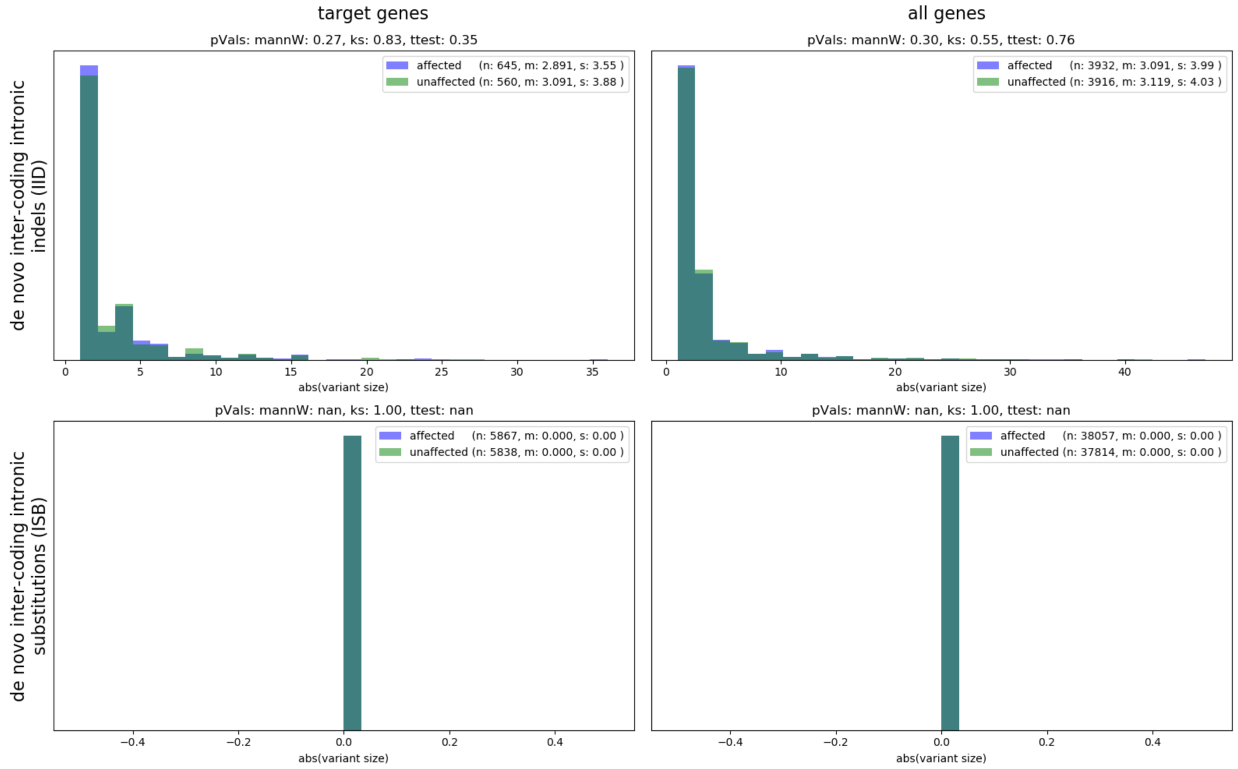

Each of the Supplementary Figures 8 to 32 corresponds to a property of *de novo* intronic events (see Supplementary Table 2 and the Supplementary Note 2 for a list and definition of the properties). For example, Supplementary Figure 8 refers to the 'variant size' property. Each of the 25 figures has four panels that correspond to four comparisons of the property for two sub-classes of observed *de novo* intronic events. The two classes of events compared in each plot are indicated with strings "affected" and "unaffected" that indicate child affected status; rows IID and ISB (y-axis) that stand for *de novo* intronic indels and substitutions; and columns "all genes" and "target genes" that indicate all genes or the subset that represent autism target genes. The numbers of events in the two classes are shown next to the class definition, and the distribution of the properties for the two classes of events are shown with the two histograms (purple vs. green) in the plot. We compare the two distributions with three different statistical tests: Mann-Whitney ("mannW") test, Kolmogorov–Smirnov ("ks") test, and t-test ("ttest"). The p-values from the three tests are shown in the title of each plot.

Supplementary Figure 9. Intron length distributions

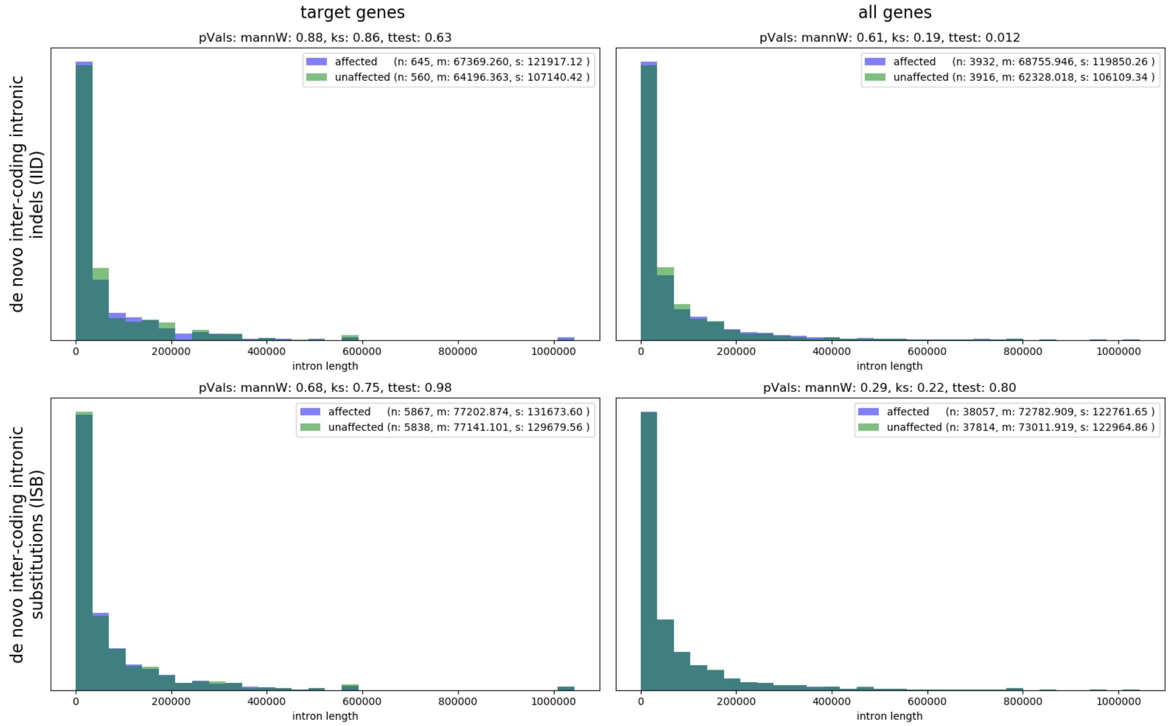

Supplementary Figure 10. Distance from splice site distributions

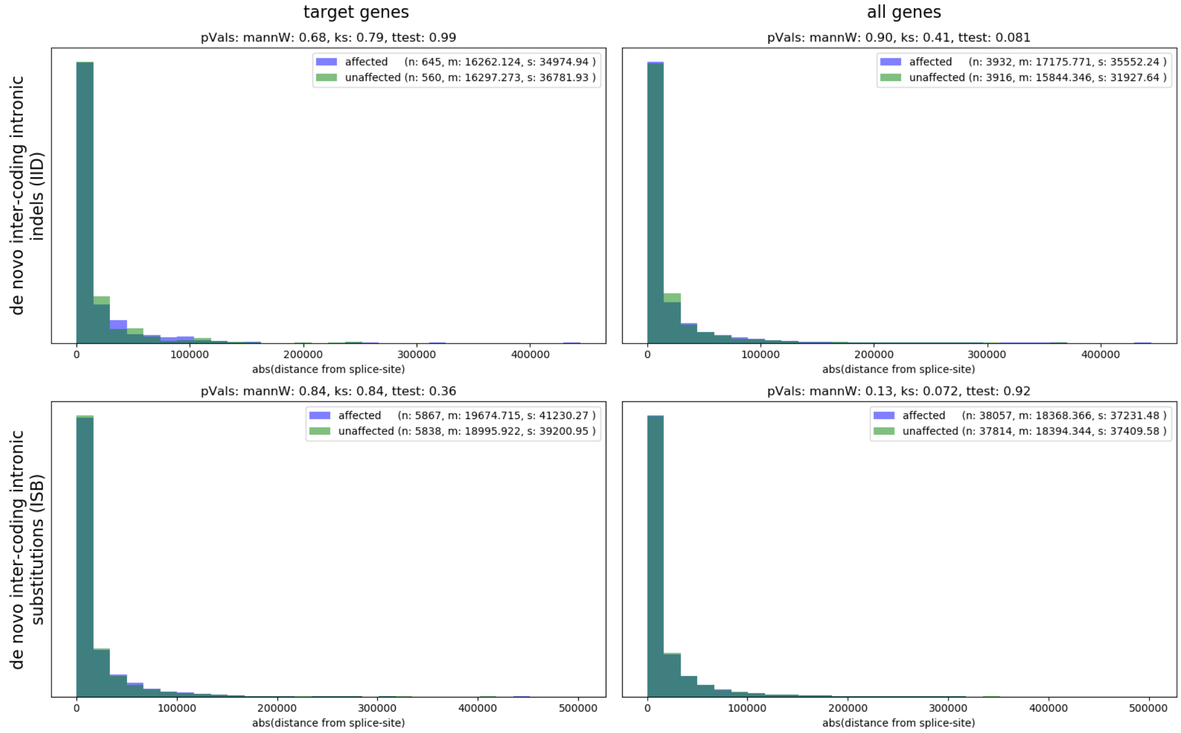

Supplementary Figure 11. ORF length distributions

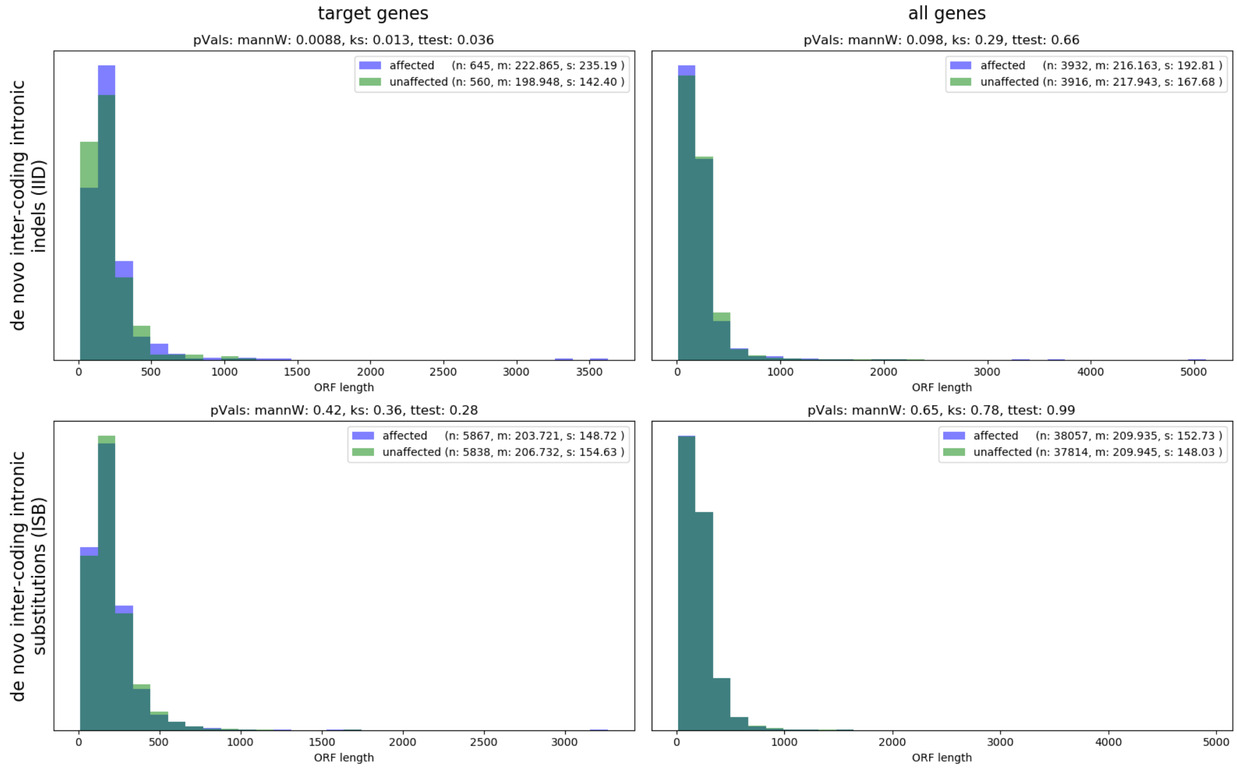

Supplementary Figure 12. SpliceAI DS_AG score distributions

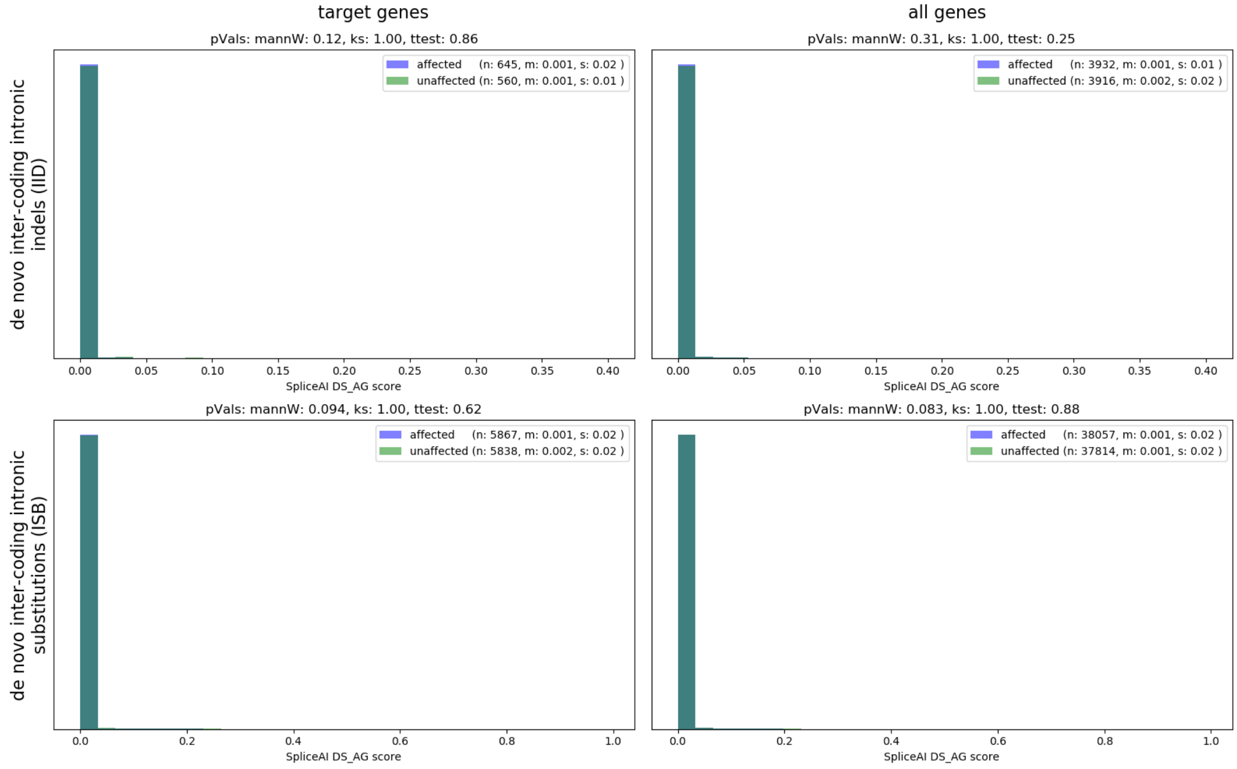

Supplementary Figure 13. SpliceAI DS_AL score distributions

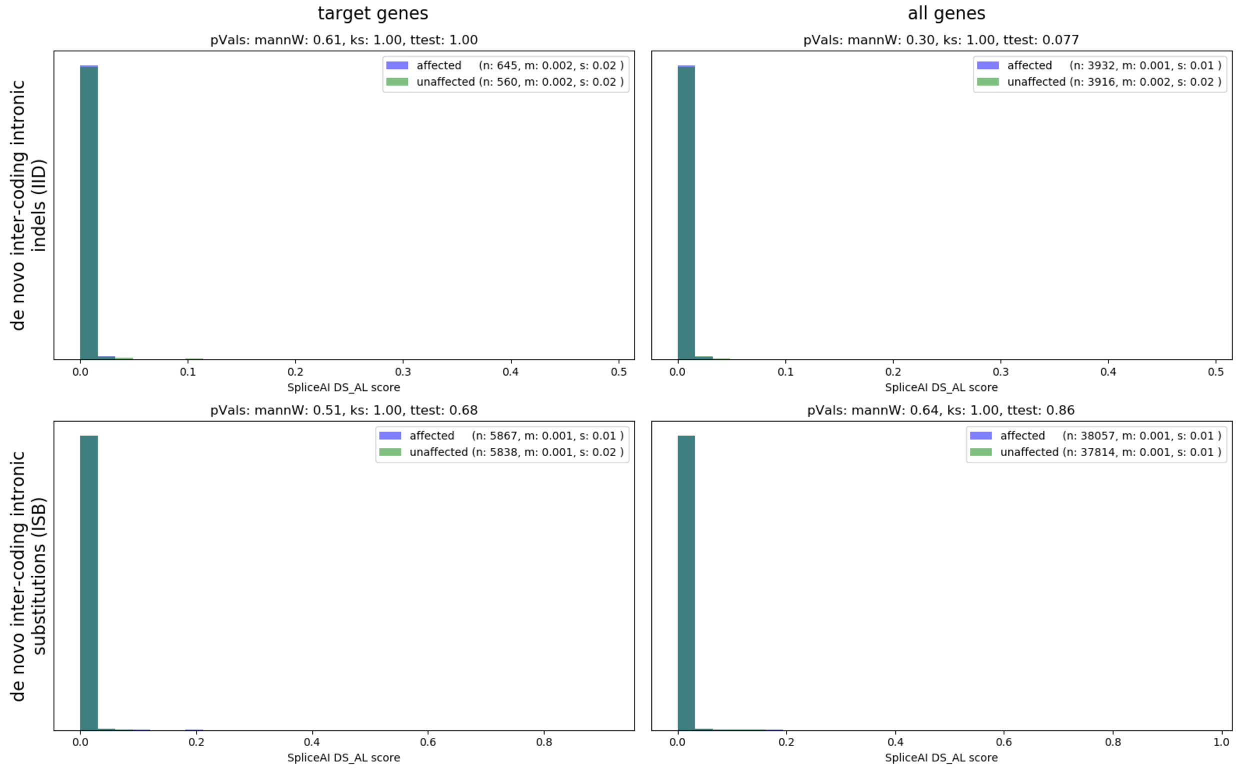

Supplementary Figure 14. SpliceAI DS_DG score distributions

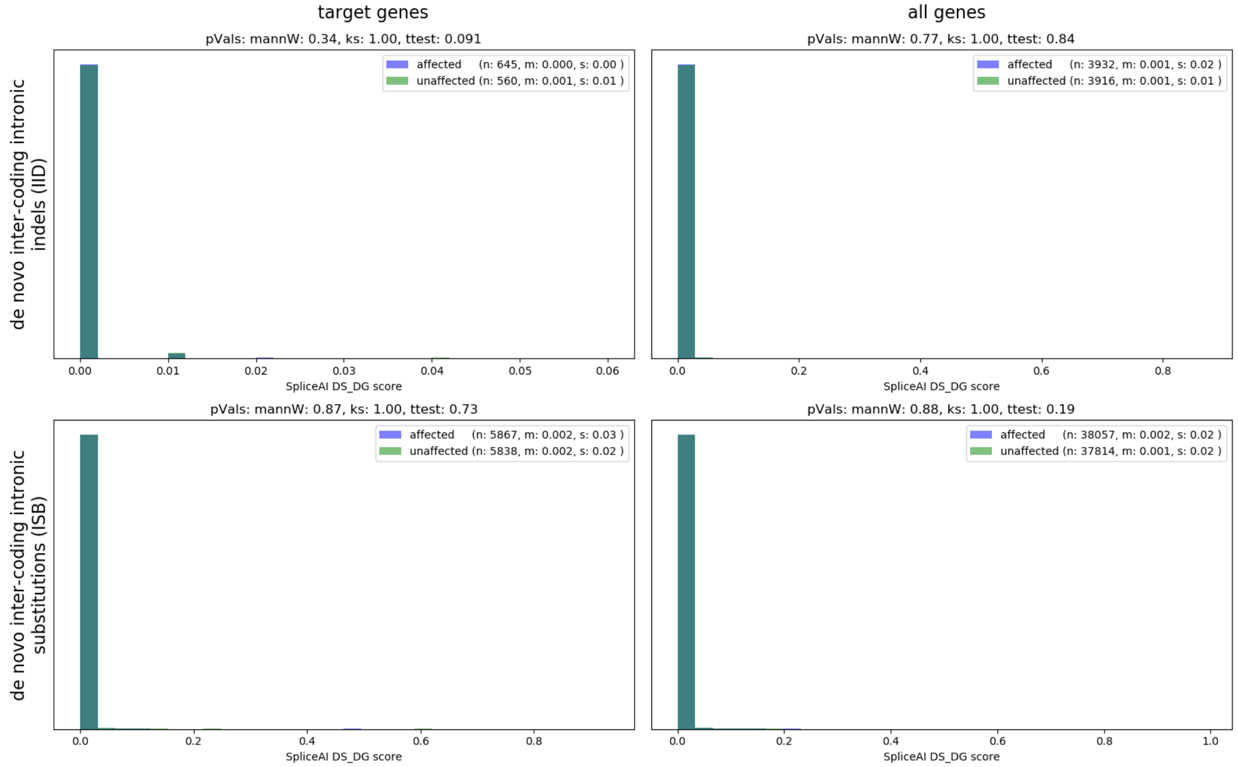

Supplementary Figure 15. SpliceAI DS_DL score distributions

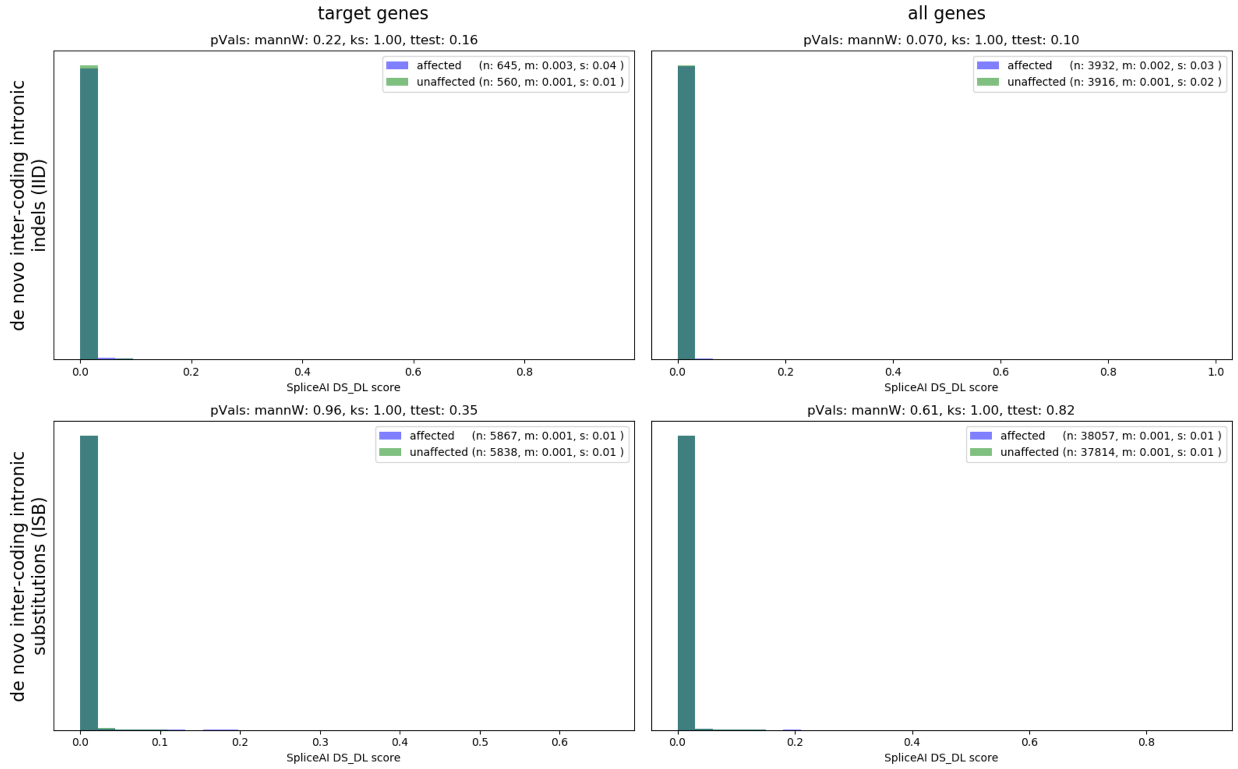

Supplementary Figure 16. SpliceAI MAX_DS score distributions

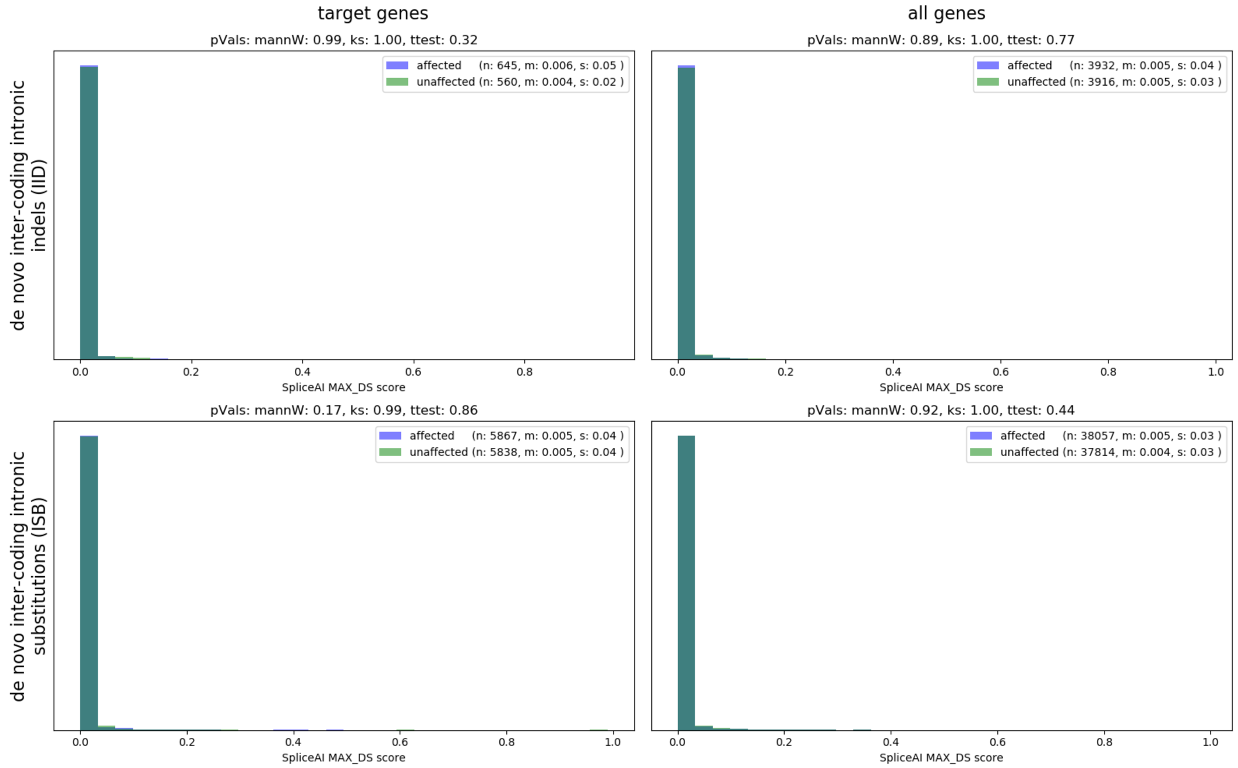

Supplementary Figure 17. Acceptor alt score distributions

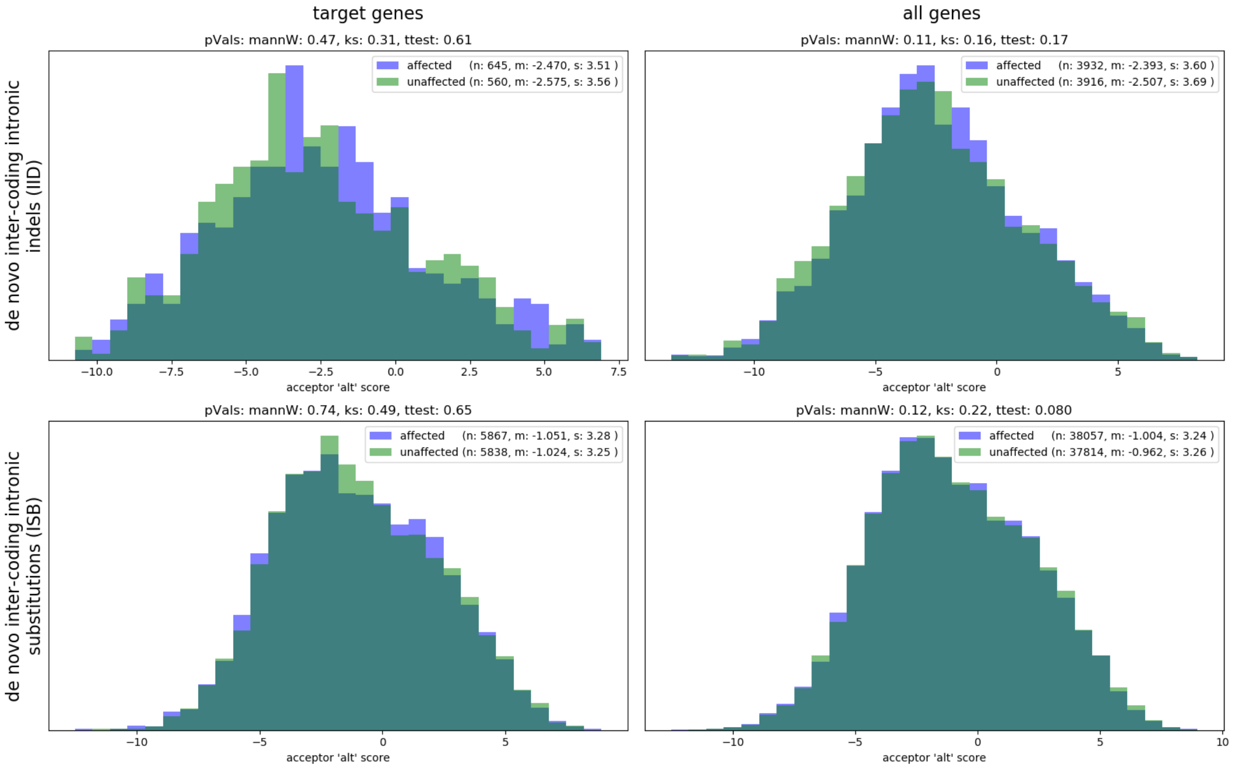

Supplementary Figure 18. Acceptor ref score distributions

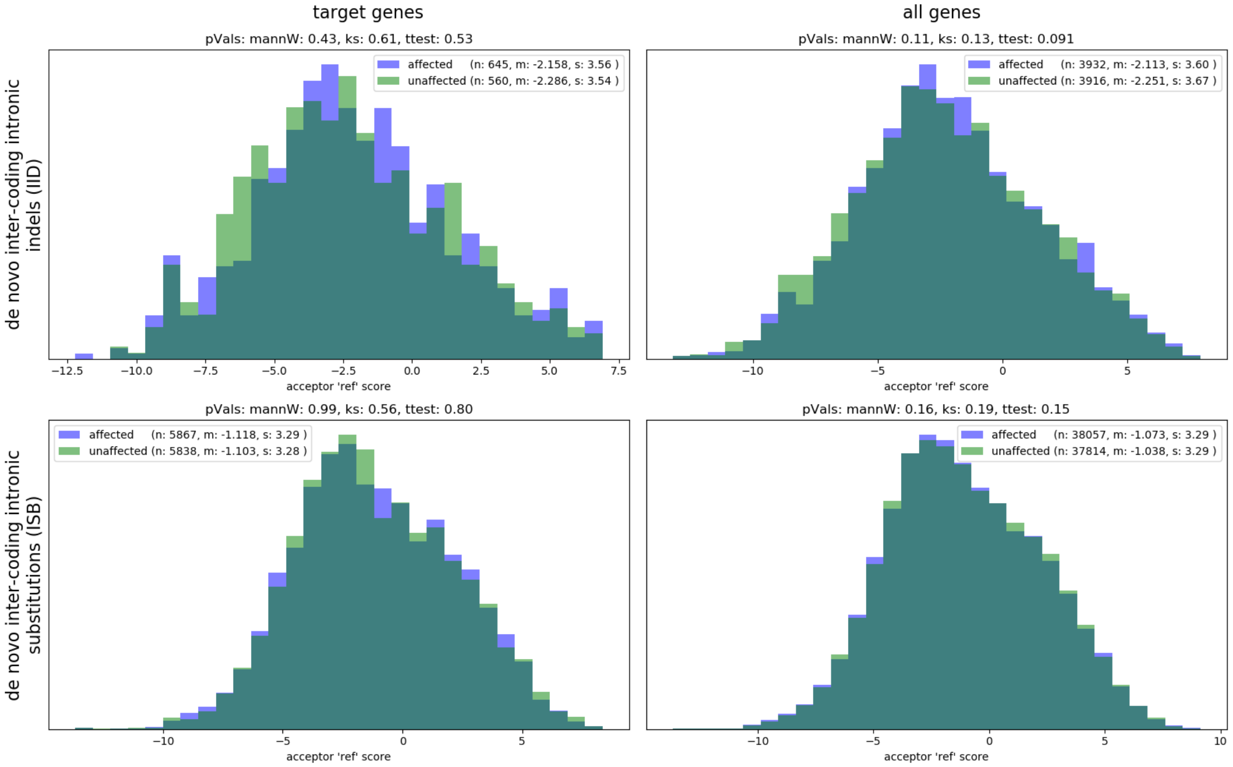

Supplementary Figure 19. Acceptor alt-ref score distributions

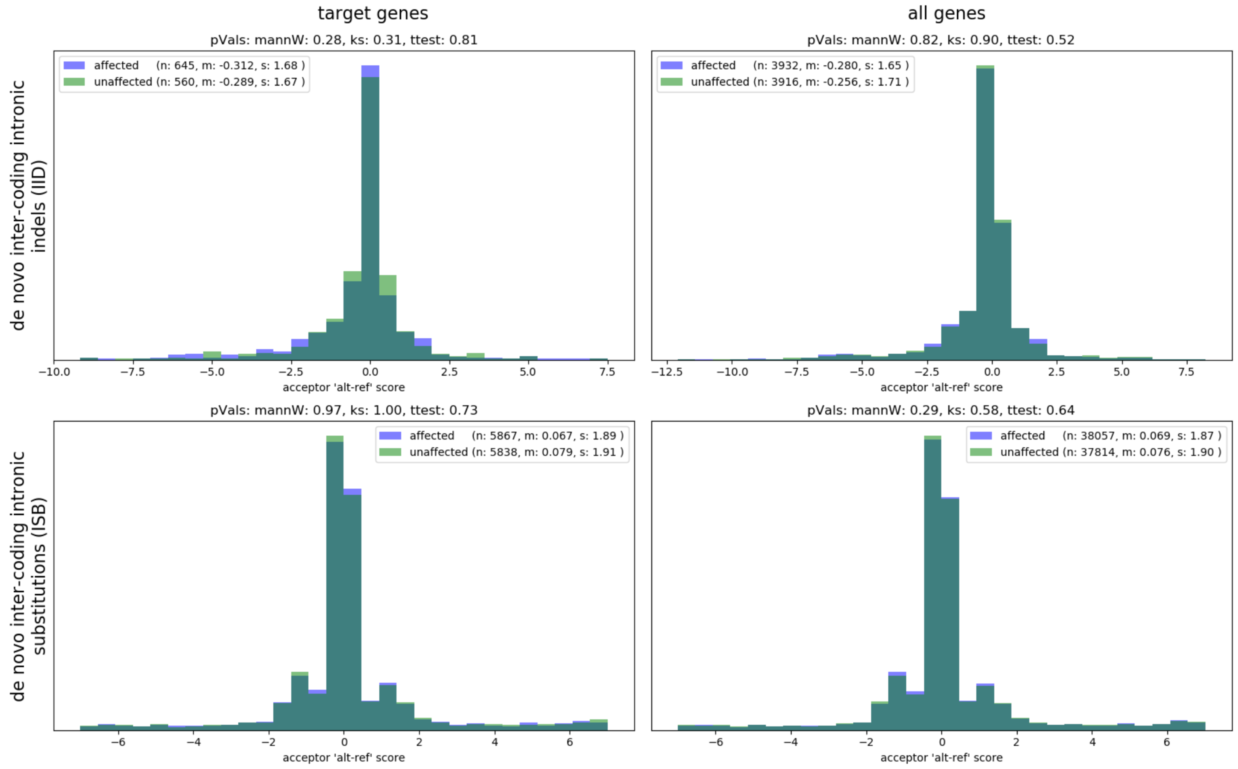

Supplementary Figure 20. Donor alt score distributions

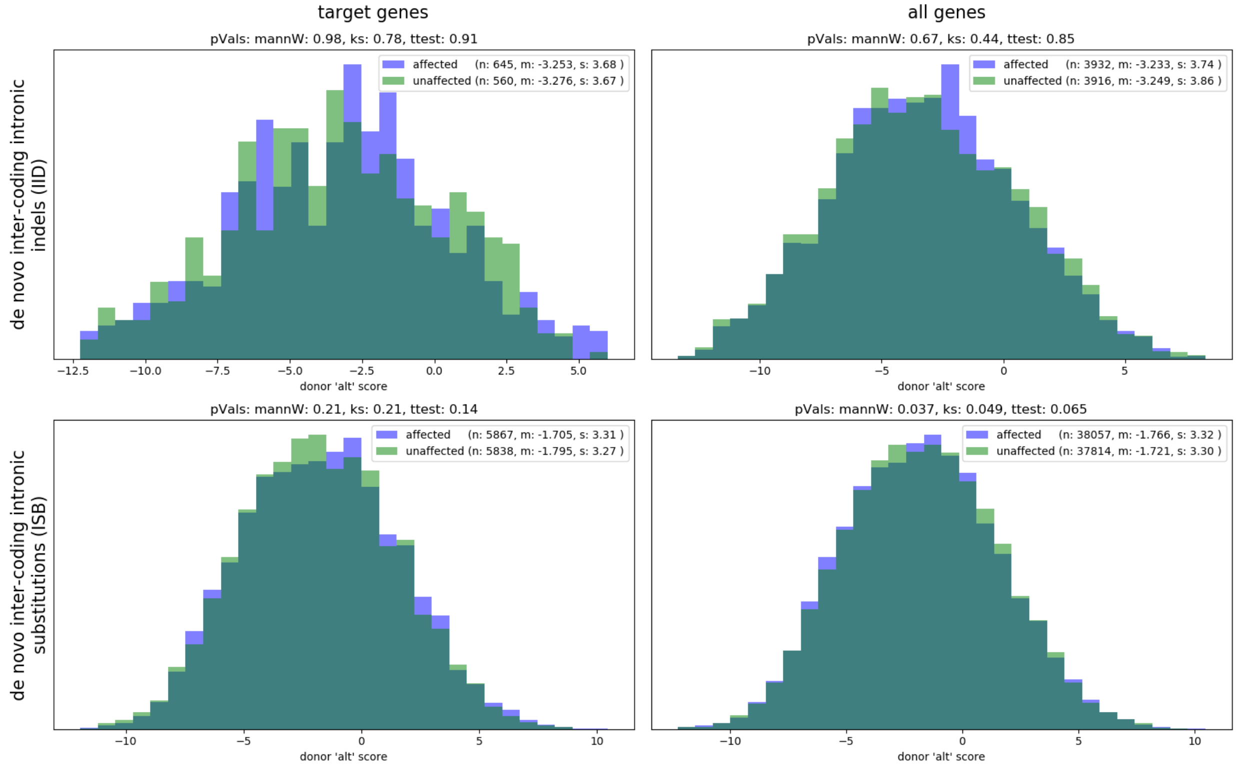

Supplementary Figure 21. Donor ref score distributions

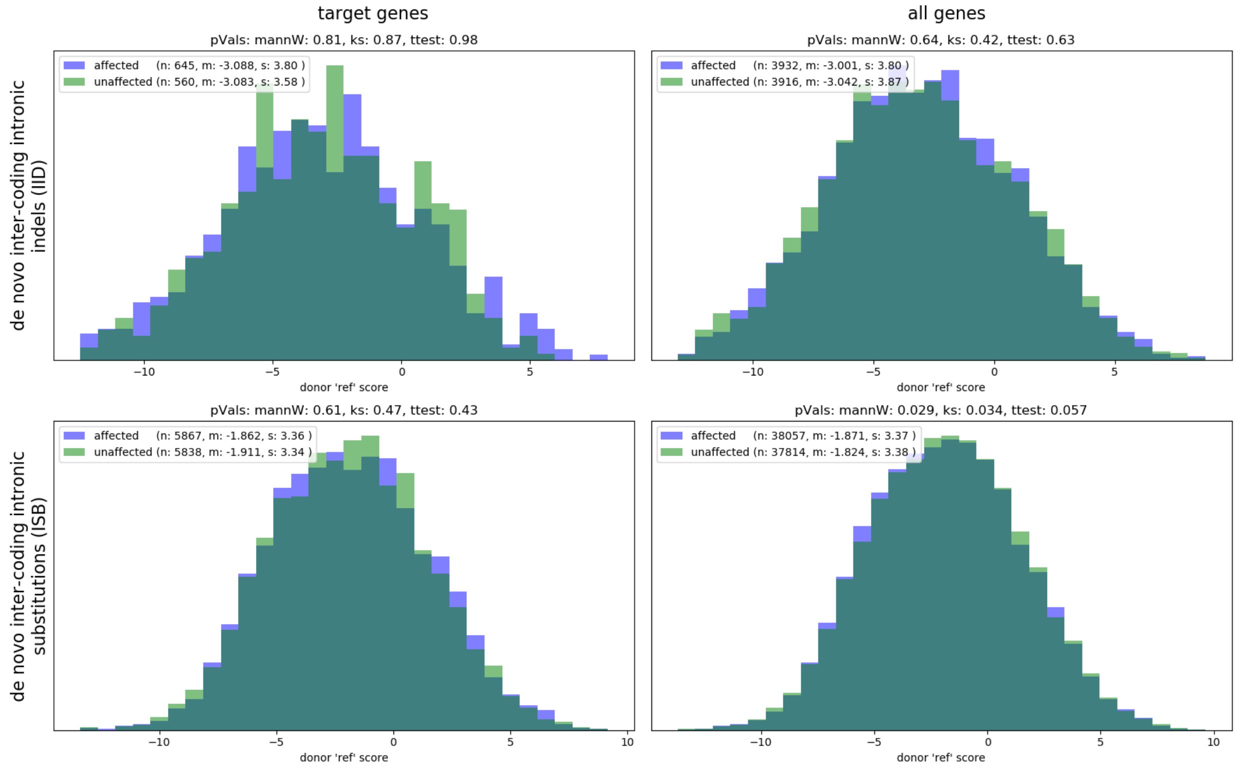

Supplementary Figure 22. Donor alt-ref score distributions

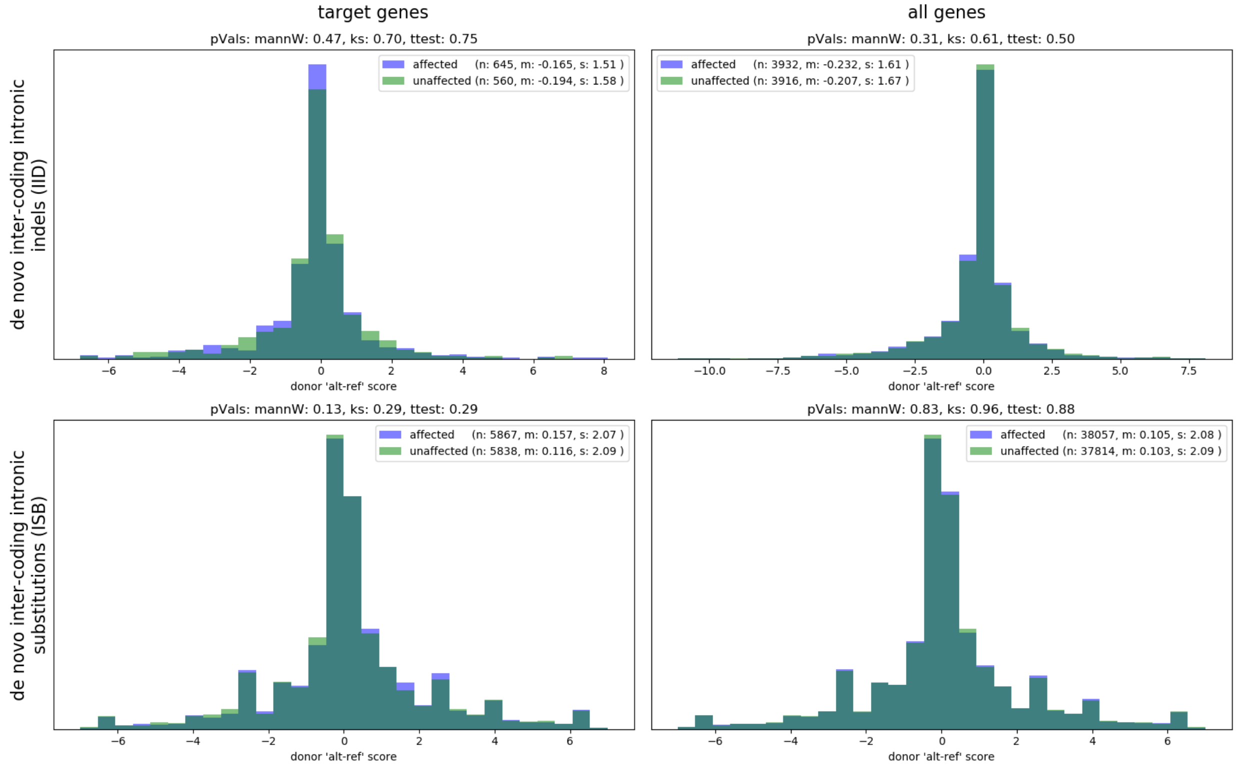

Supplementary Figure 23. phylop, 100 vertebrates score distributions

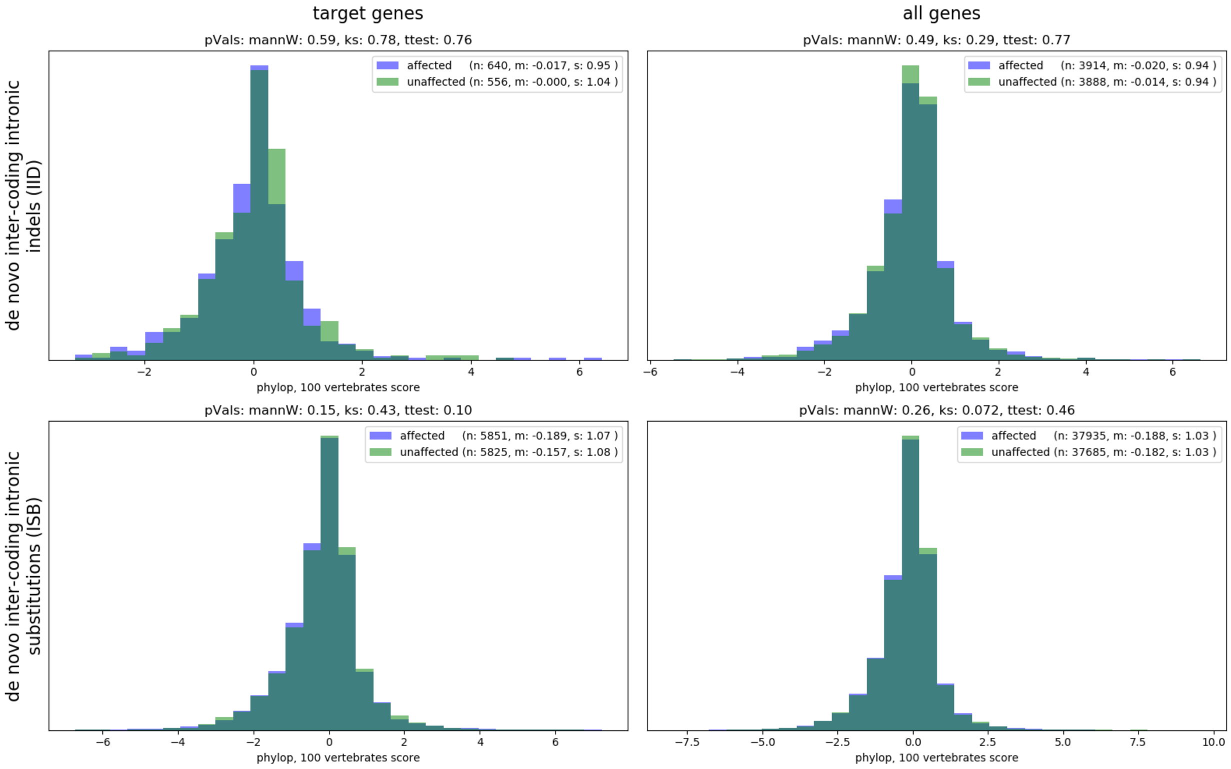

Supplementary Figure 24. phylop, 30 vertebrates score distributions

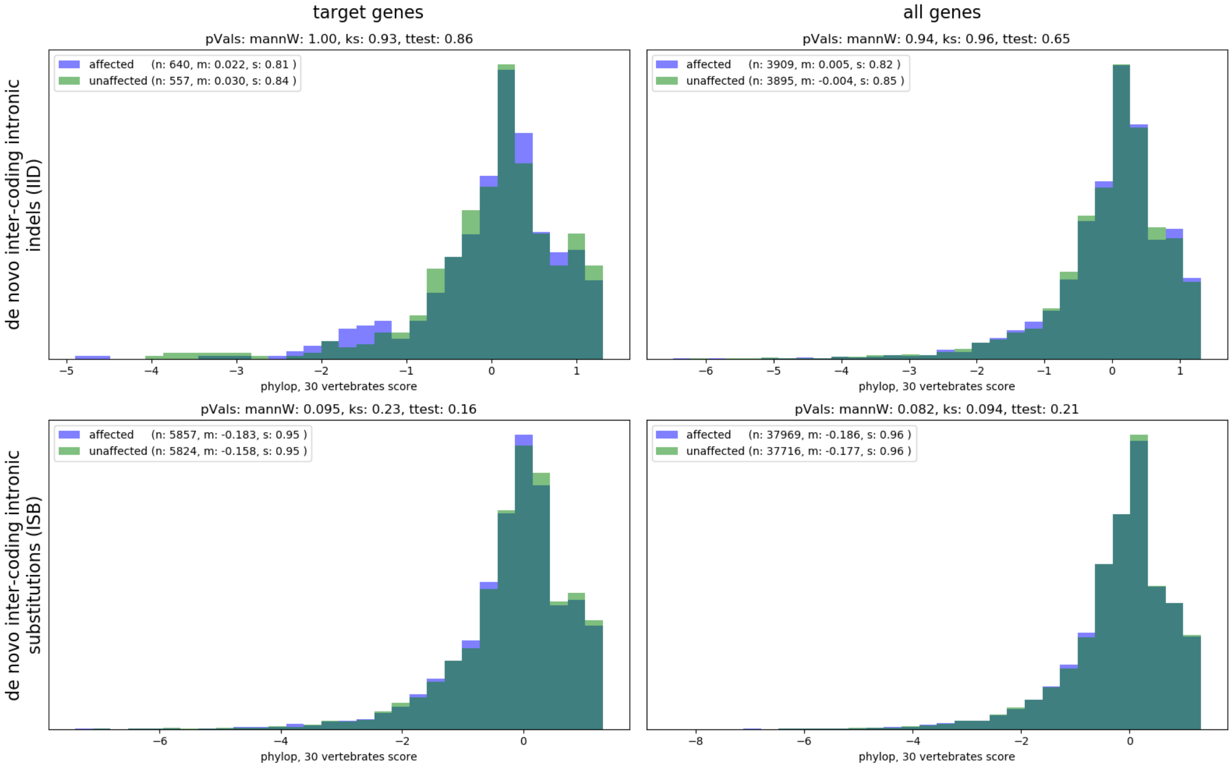

Supplementary Figure 25. phylop, 20 vertebrates score distributions

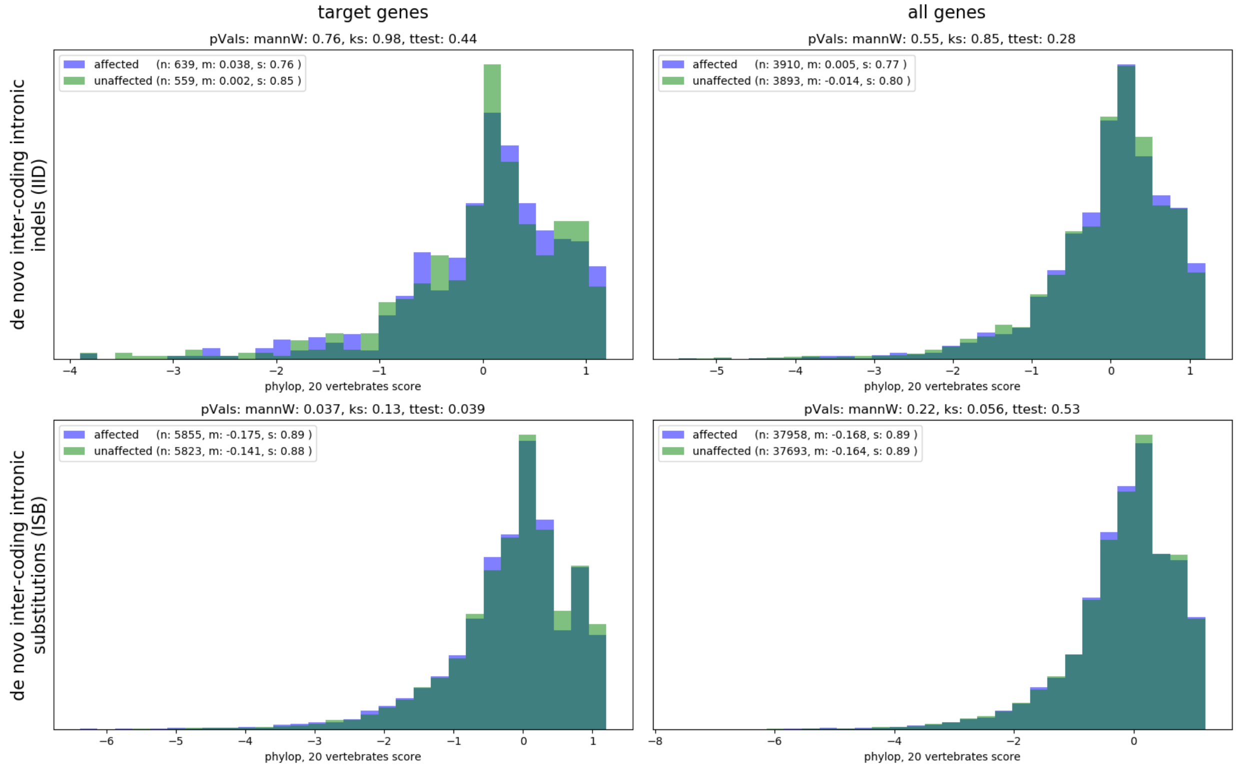

Supplementary Figure 26. phylop, 7 vertebrates score distributions

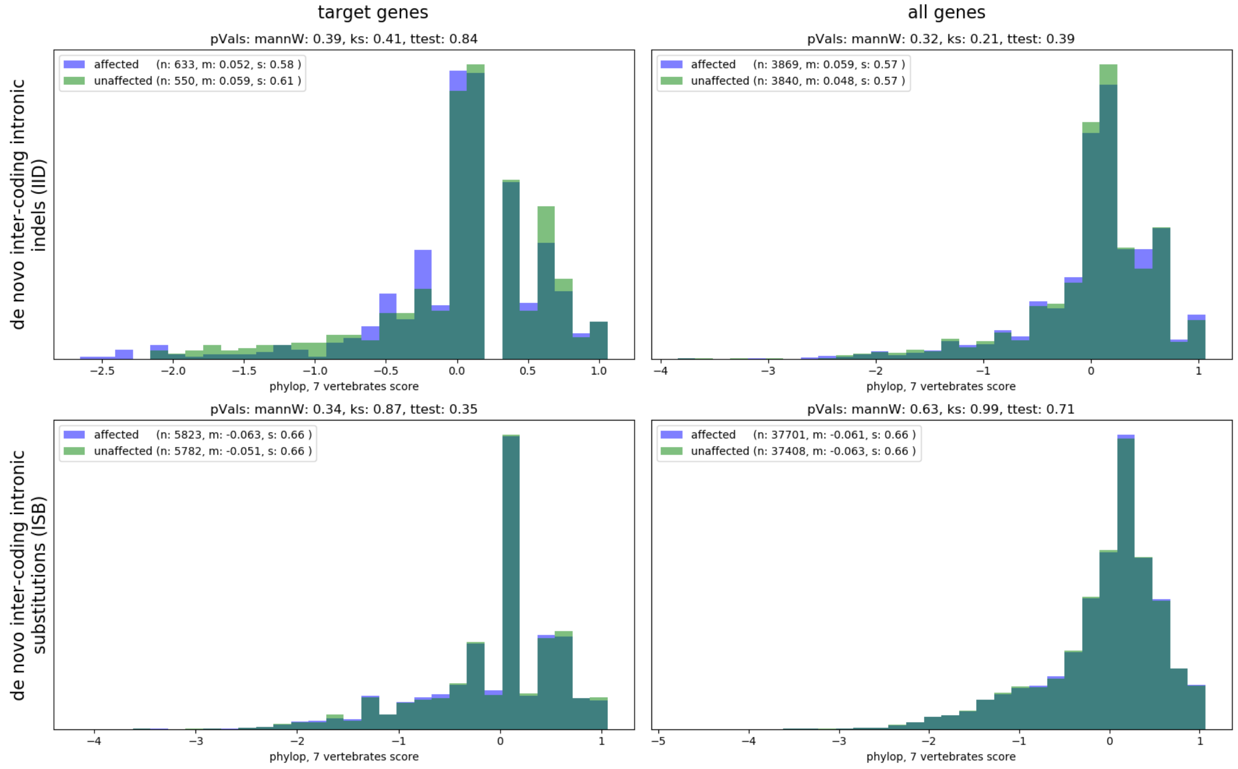

Supplementary Figure 27. phastCons, 100 vertebrates score distributions

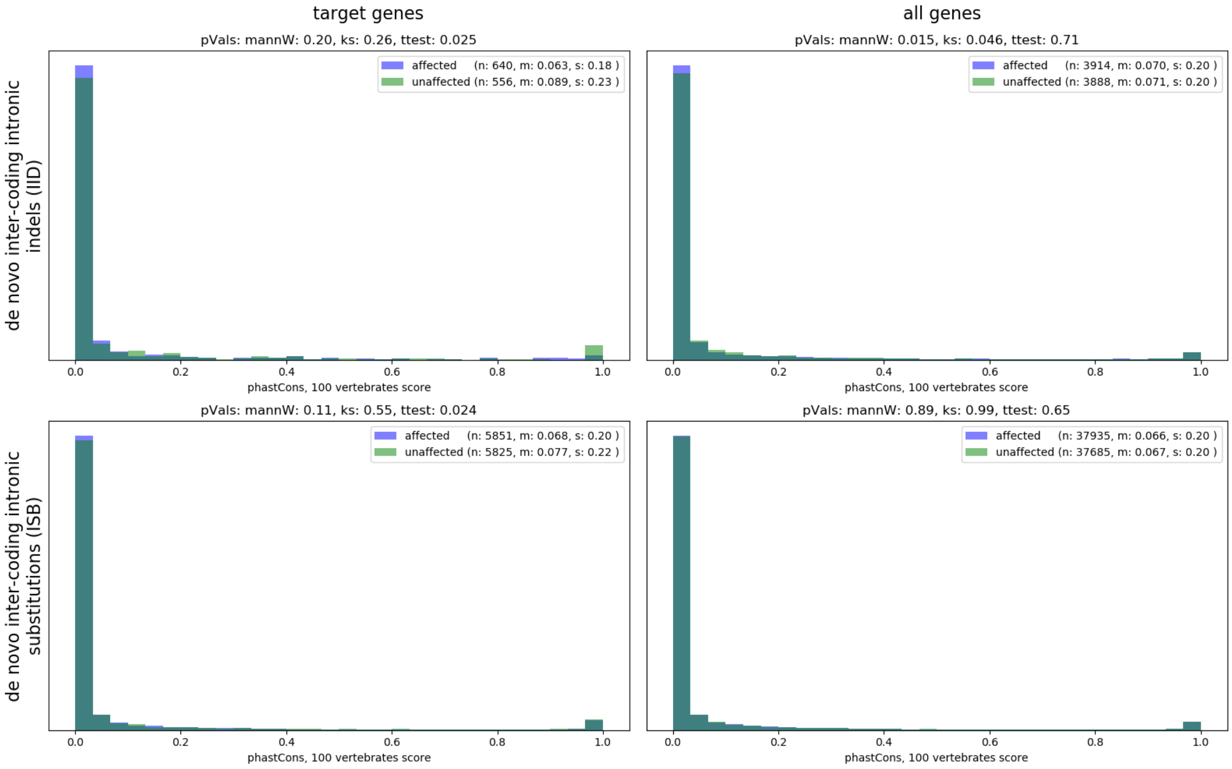

Supplementary Figure 28. phastCons, 30 vertebrates score distributions

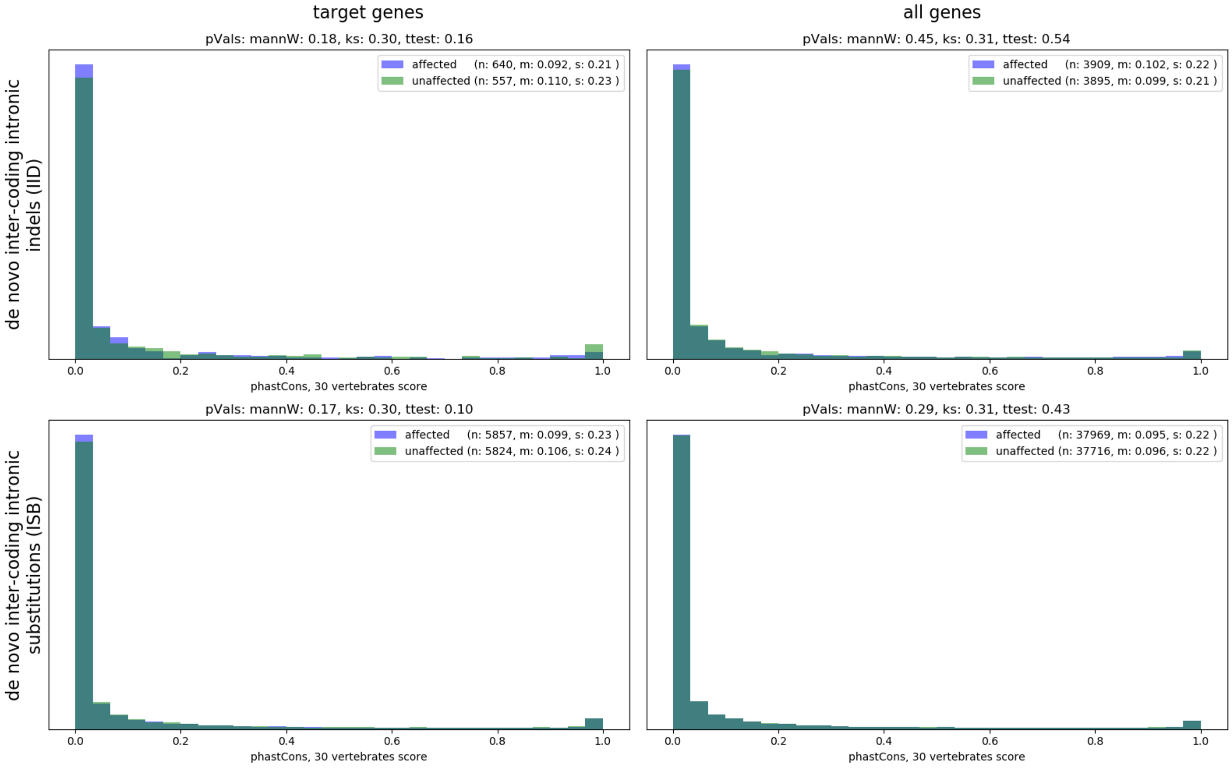

Supplementary Figure 29. phastCons, 20 vertebrates score distributions

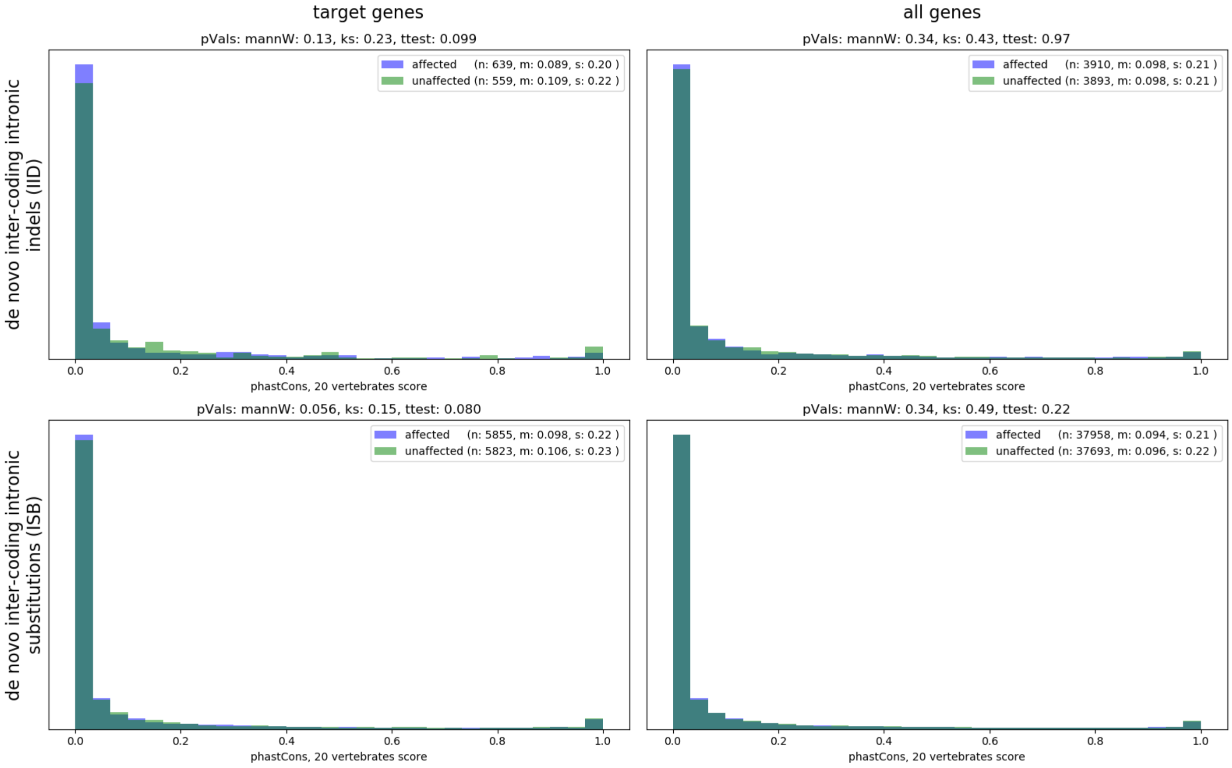

Supplementary Figure 30. phastCons, 7 vertebrates score distributions

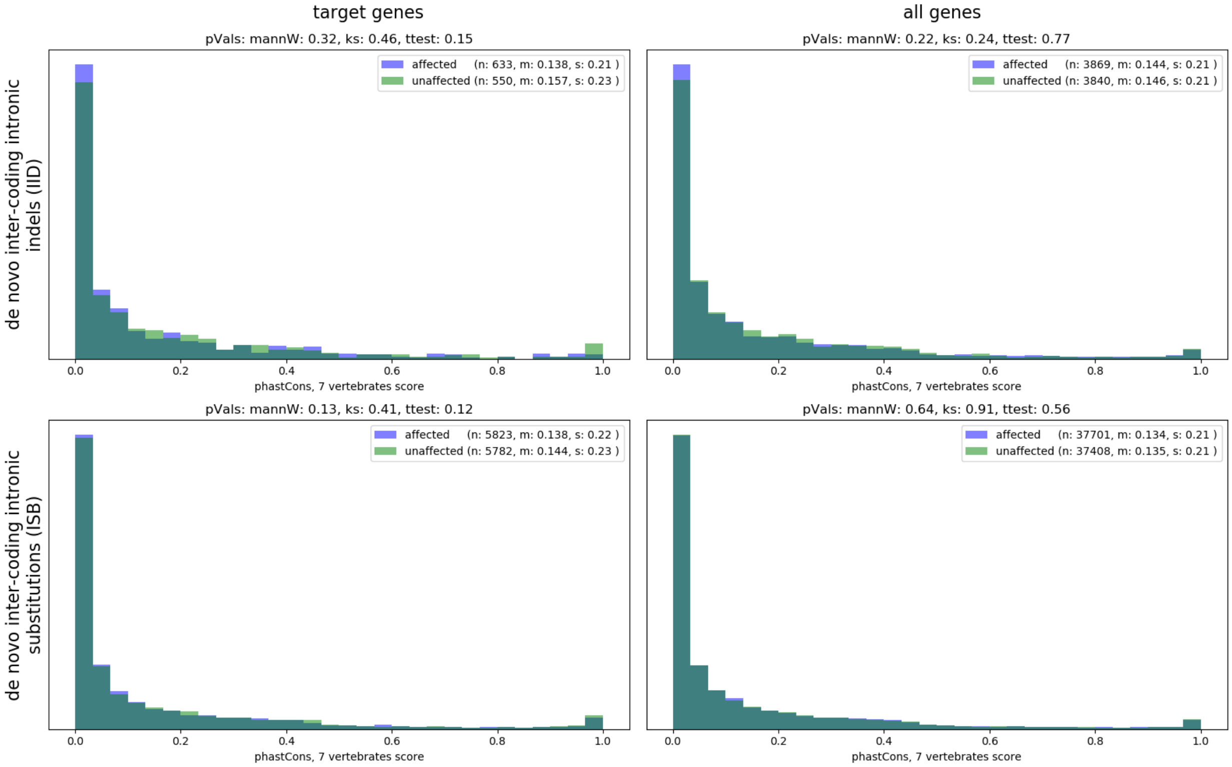

Supplementary Figure 31. CADD score distributions

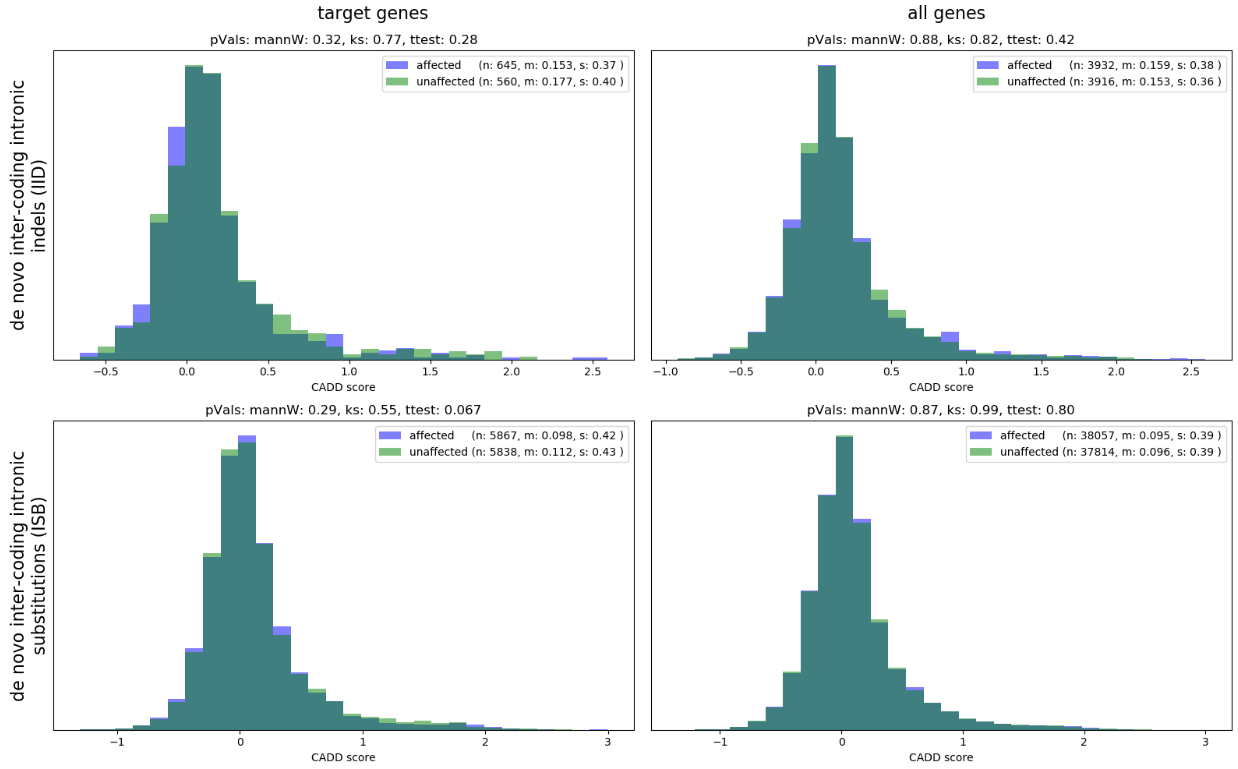

Supplementary Figure 32. Minimum property rank distributions

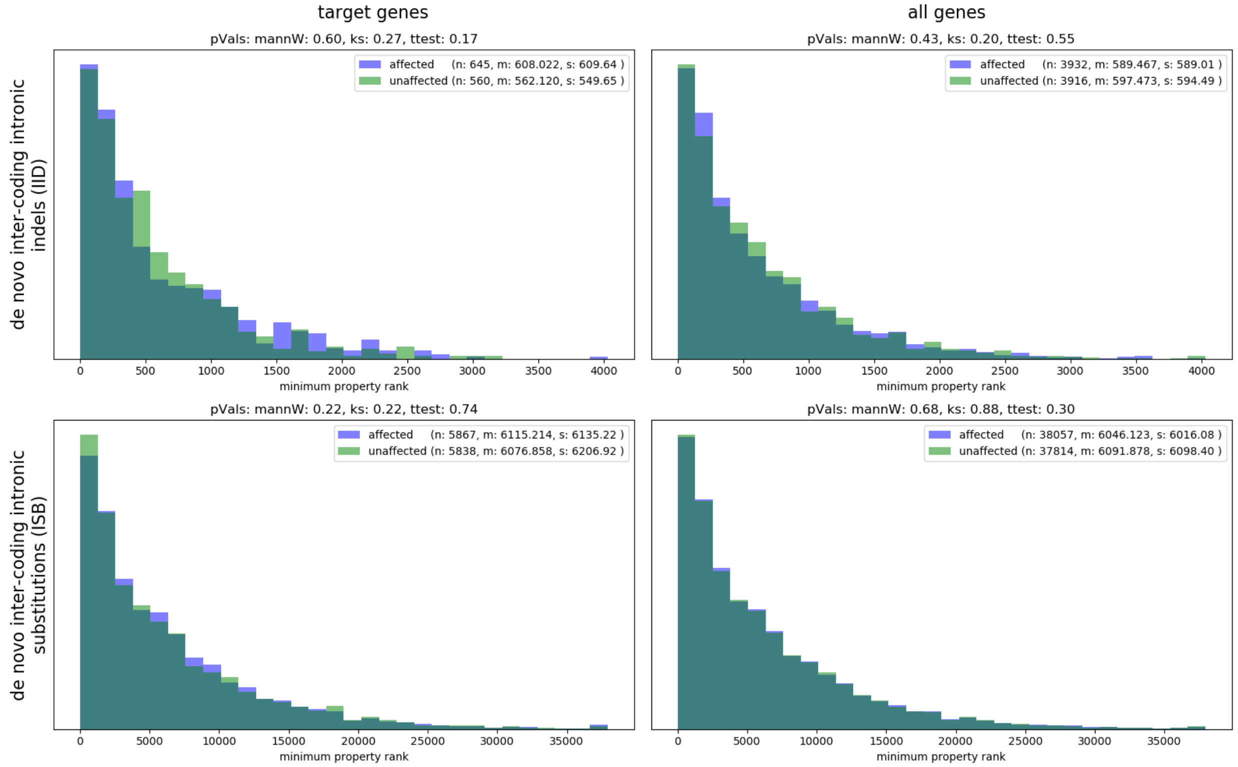

Supplementary Figure 33. Splice-site model

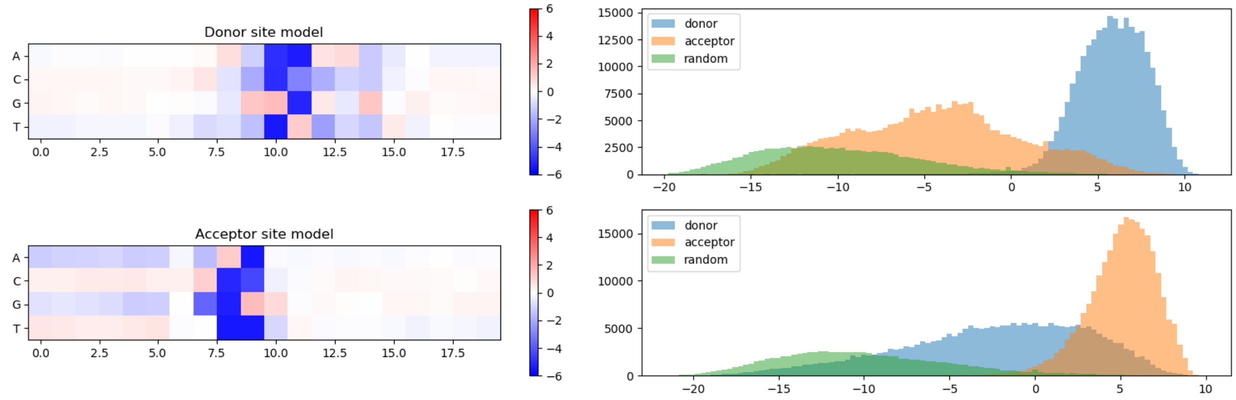

Weights for the Donor ($w_{pn}^{\mathcal{D}}$) models are plotted in the top left panel and the weights for the Acceptor ($w_{pn}^{\mathcal{A}}$) model are plotted in the bottom left panel (see Supplementary Note 2). In the right top panel, we plot the distribution of the position-specific donor splice-site scores for three sets of genomic locations: annotated donor-splice sites (blue), annotated acceptor splice-sites (orange) and random intronic positions (green). Similarly, in the right bottom panel, we plot the distributions of the position-specific acceptor splice-site scores for the same three sets of locations.

Supplementary Figure 34. An example of acceptor splice-site sequence score

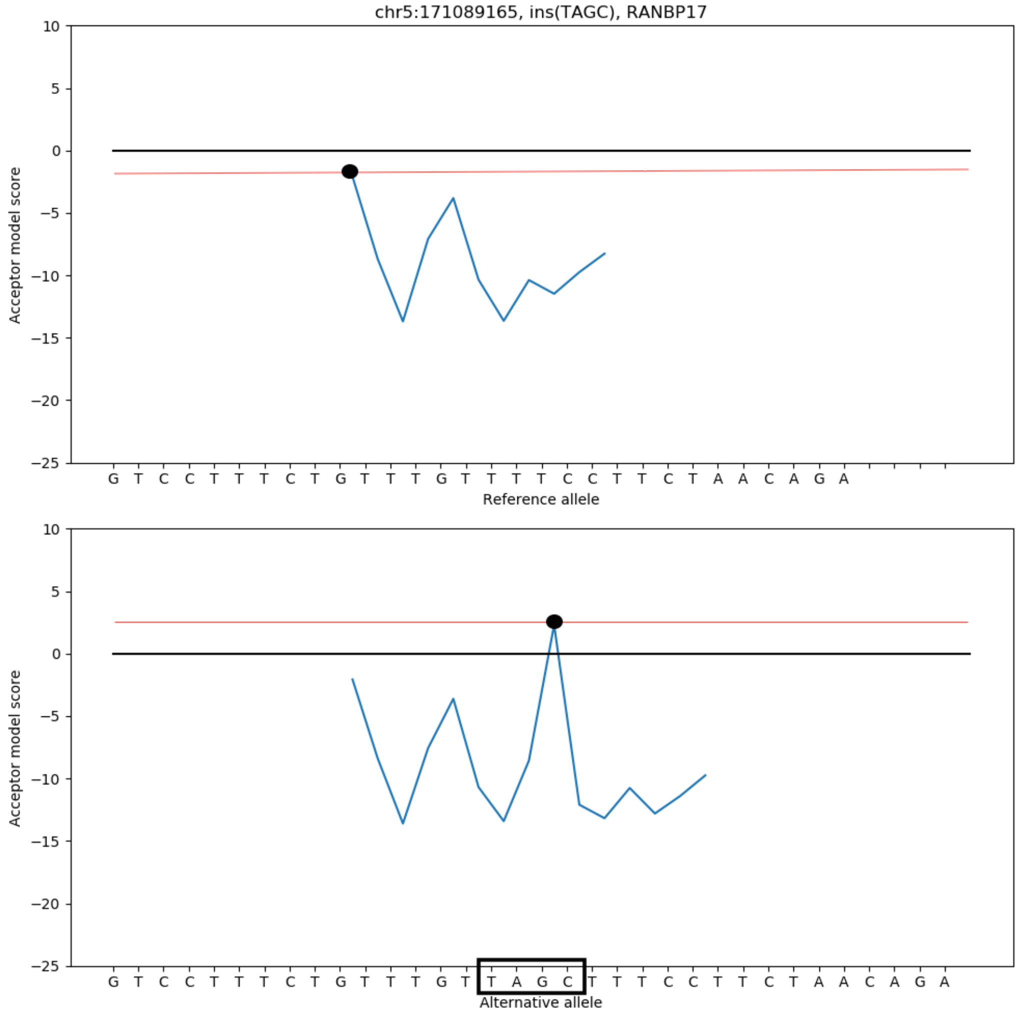

An example of the acceptor sequence score for the *de novo* intronic indel: ins(TAGC) found in chromosome 5, position: 171,189,165 in gene *RANBP17* is shown. The red line in the top panel depicts the acceptor position-specific score (*y*-axis) for the reference allele; the large black dot shows the position and the score for the maximum position-specific score that is used as the acceptor splice-score (red line) for the reference allele. Similarly, the bottom panel shows the position-specific splice-site scores and the splice-site score for the alternative allele after the insertions has been introduced. The *x*-axis shows each nucleotide in the sequence context for that splice site (Supplementary Note 2). For example, the acceptor splice-site sequence context for the reference allele (top panel) is GTCCTTTCTGTTTGTTTTCC for the splice site position corresponding to the large black dot.

Supplementary Figure 35. HMM *de novo* CNV finder

A) Hidden Markov Model parameters

Transition probabilities

The transitions matrix from bin i to bin i+1,

$$T_i = T^{d_i},$$

where d_i is the distance in bp of the middle positions of bin i and bin i+1 and

T = (rows: state from; columns: state to)

state from \ state to	0	1	2	3	4
0	b	d	c	d	d
1	d	b	c	d	d
2	e	e	a	e	e
3	d	d	c	b	d
4	d	d	c	d	b

The transition matrix parameters τ=(a, b, c, d, e) represent the probabilities of the following events occurring between two neighboring genomic positions, where state 2 is called ground, and the other states are called out-of-ground (OOG):

- a - staying in ground state;
- b - staying in the same OOG state;
- c - transitioning from OOG to ground;
- d - transitioning from OOG to a different OOG;
- e - transitioning from ground to OOG.

Initial state probabilities

The initial state distribution,

$$T_0 = T^{\infty}.$$

Emission probabilities

$$E(c_{pb}|S) \propto \text{Poisson}(\lambda_{pb} S^*),$$

where $S \in \{0,1,2,3,4\}$, c_{pb} are the observed counts for person p and bin b, λ_{pb} are the per person and bin specific rate parameters, and $S^* = \max(0.1, S)$.

B) SVD normalization

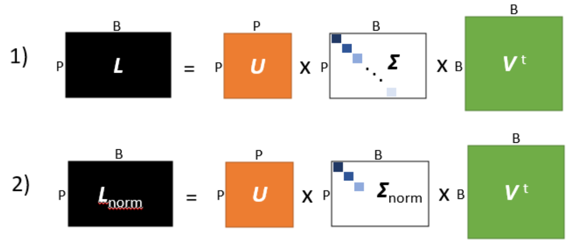

C) HMM/SVD EM algorithm

INPUT: ***C***, transition matrix parameters (τ) initial values
 τ := initial values
 H_0 := (2 for all p and b)
 for i in 1,2,3:
  L_{i-1} := C / H_{i-1} (element wise, with 0 elements of ***H*** replaced with 0.1)
  L_i := SVD normalization of L_{i-1}
  repeat until H_i does not change:
   H_i := Viterbi(***C***, L_i, τ)
   τ := maximum likelihood estimates of τ based on H_i
OUTPUT: H_3, L_3

D) *De novo* CNV finder

The trio specific subset of *H*

mother	2	2	2	2	2	**1**	**1**	**0**	**1**	2
father	2	2	2	2	2	2	2	2	2	2
child	2	**3**	**3**	**4**	2	2	**1**	**1**	**1**	2

Polar representation

mother	0	0	0	0	0	**-1**	**-1**	**-1**	**-1**	0
father	0	0	0	0	0	0	0	0	0	0
child	0	**1**	**1**	**1**	0	0	**-1**	**-1**	**-1**	0

de novo candidate

We show the four main components of our HMM *de novo* CNV finder algorithm. The algorithm takes a count P by B matrix ***C*** representing the counts for P individuals and B bins. The detailed description of how bins are defined and reads counted can be found in the “HMM *de novo* CNV finder” section above.

We model the counts for the bins of each individual with a Hidden Markov Model (HMM) with length equal to B, where the possible values of the hidden states S are 0, 1, 2, 3 and 4 representing the copy number of the genomic region related to the particular bin.

Panel A describes in detail the parameters of HMM models. The parameters used in the transition and initial probabilities (τ) are shared across individuals, whereas the emission parameters, λ_{pb}, used in the Poisson emission probability model are specific for each individual and are organized in a P by B rate matrix ***L***.

Panel B outlines the SVD based normalization procedure we apply for the rate matrix ***L***. First, we use Singular Value Decomposition for the matrix ***L*** as implemented in the Python's SciPy library. Second, we compute a normalized matrix $\boldsymbol{L}^{norm}$ by using only the 3 components with the maximum eigen values.

Panel C shows the HMM/SVD EM algorithm, an iterative Expectation-Maximization procedure to simultaneously identify the maximum likelihood states for the hidden variables for each individual and bin (matrix ***H***) and the rate matrix ***L***. The procedure uses the Viterbi algorithm[13] for finding the maximum likelihood states of the hidden variables separately for the HMMs associated with each individual. The maximum likelihood estimates for the transition parameters (τ) are based on straightforward counting of the transitions among the states in the ***H*** matrix.

Panel D outlines the procedure we used to identify *de novo* CNV candidates from the state matrix ***H*** produced by HMM/SVD EM algorithm. We analyze separately all subsets of 3 rows from the matrix ***H*** corresponding to the mother, father, and child trios available among the analyzed population. First, we summarize the trio subsets of ***H*** into what we call polar representation. In polar representation, the ground state (2 for autosomes and 2, 1 or 0 for the X and Y chromosome depending on gender) is represented by 0, states larger than the ground state are represented by 1 and states smaller than the ground are represented by -1. Then, the polar representation matrix is split by bin intervals of constant polar states for all members of the trio (the differently colored sections). Finally, bin intervals in which the two parents have polar values of 0 and for which the child has a polar value of 1 or -1 are listed as candidate *de novo* duplications and deletions, respectively.

Supplementary Tables

Supplementary Table 1. Indels and Substitutions in Peripheral Regions

			SSC unaffected		SSC unaffected						
Set	gene number	event type	functional number	normalization number	functional number	normalization number	expected functional number	delta	pval	AD	PC
all genes	19,512	indel	799	5,859	781	5,768	786.6	-5.6	0.55	-0.30% (-5.06-4.11)	-0.7% (-12.8-9.3)
autism LGD targets	748	indel	68	5,859	73	5,768	66.9	6.1	0.32	0.32% (-1.01-1.65)	8.3% (-30.4-36.6)
all NDD LGD targets	1,521	indel	120	5,859	122	5,768	118.1	3.9	0.40	0.21% (-1.41-1.84)	3.2% (-25.2-24.7)
autism missense targets	3,560	indel	212	5,859	195	5,768	208.7	-13.7	0.75	-0.73% (-3.00-1.45)	-7.0% (-32.1-12.2)
autism synonymous targets	1,570	indel	100	5,859	99	5,768	98.4	0.6	0.47	0.03% (-1.49-1.48)	0.6% (-32.9-24.5)
all genes	19,512	sub	6,748	58,274	6,903	59,340	6,871.4	31.6	0.41	1.69% (-11.12-14.95)	0.5% (-3.1-4.0)
autism LGD targets	748	sub	532	58,274	508	59,340	541.7	-33.7	0.84	-1.80% (-5.25-1.96)	-6.6% (-20.4-6.8)

Legend: This table has an identical structure to Table 5. The difference is that here we tabulate the numbers of peripheral indels and substitutions instead of the intercoding intronic indels (IID) and substitutions (ISB). See the legend of Table 5 for further details.

Supplementary Table 2. Results of the functional analysis

property	Supplementary Figure number	tests for difference between affected and unaffected children of *de novo* inter-coding intronic indels (IID) in		tests for difference between affected and unaffected children of *de novo* inter-coding intronic substitutions (ISB) in	
		target genes	all genes	target genes	all genes
variant size	8	0.27	0.30		
intron length	9	0.88	0.61	0.68	0.29
distance from splice-site	10	0.68	0.90	0.84	0.13
ORF length	11	0.0088	0.098	0.42	0.65
**SpliceAI scores**					
SpliceAI DS_AG score	12	0.12	0.31	0.094	0.083
SpliceAI DS_AL score	13	0.61	0.30	0.51	0.64
SpliceAI DS_DG score	14	0.34	0.77	0.87	0.88
SpliceAI DS_DL score	15	0.22	0.07	0.96	0.61
SpliceAI MAX_DS score	16	0.99	0.89	0.17	0.92
**Simple Splice Model scores**					
acceptor 'alt' score	17	0.47	0.11	0.74	0.12
acceptor 'ref' score	18	0.43	0.11	0.99	0.16
acceptor 'alt-ref' score	19	0.28	0.82	0.97	0.29
donor 'alt' score	20	0.98	0.67	0.21	0.037
donor 'ref' score	21	0.81	0.64	0.61	0.029
donor 'alt-ref' score	22	0.47	0.31	0.13	0.83
**Conservation scores**					
phylop, 100 vertebrates score	23	0.59	0.49	0.15	0.26
phylop, 30 vertebrates score	24	1.00	0.94	0.095	0.082
phylop, 20 vertebrates score	25	0.76	0.55	0.037	0.22
phylop, 7 vertebrates score	26	0.39	0.32	0.34	0.63
phastCons, 100 vertebrates score	27	0.20	0.015	0.11	0.89
phastCons, 30 vertebrates score	28	0.18	0.45	0.17	0.29
phastCons, 20 vertebrates score	29	0.13	0.34	0.056	0.34
phastCons, 7 vertebrates score	30	0.32	0.22	0.13	0.64
CADD score	31	0.32	0.88	0.29	0.87
**Min Rank Aggregated score**					
minimum property rank	32	0.6	0.43	0.22	0.68

Legend: We tested each of the 25 properties listed in column ‘property’ for their ability to separate subsets of the different classes of *de novo* intronic events identified through whole-genome data from 1,869 affected and 1,874 unaffected children. The classes are defined by the *de novo* intronic event type (IID for *de novo* intronic indel or ISB for *de novo* intronic substitution), the affected ‘status’ of the child carrying the *de novo* events (‘affected‘ or ‘unaffected’), and by the class of the gene targeted by the event (‘all genes’ or ‘target genes’ for the set of 1,521 autism genes that were targeted by *de novo* LGD mutations in children diagnosed with a neurodevelopmental disorder).

The first four properties refer to variant size, distance to the nearest splice-site ('distance from splice site'), intron and ORF length in base pairs. The next five properties refer to spliceAI scores[8]. The next six properties refer to our novel splice-site model scores that consist of two main categories: acceptor and donor sites that are subdivided in three sub scores: alternative alleles ('alt'), reference alleles ('ref'), and the difference between 'alt' and 'ref' scores ('alt-ref'). The next nine properties refer to conservation scores that are based on phyloP and phastCons scores for primates, placental mammals and vertebrates and CADD scores. The last property refers to our 'min-rank' score for every *de novo* event that combined the previous 24 property scores separately for *de novo* inter-coding intronic indels (IID) and *de novo* inter-coding intronic substitutions (ISB). See the Supplementary Note 2 for more details.

Column 'Supplementary Figure number' lists the corresponding Supplementary Figure number showing distributions of the property in the different classes of events.

Four different tests for a pair of classes are performed for each property using Mann-Whitney test and resulting p-values are listed in the columns 'target genes', and 'all genes' separately for IID and ISB. Each of the four Mann-Whitney tests compares the distribution of the corresponding property for the events in the affected children to the distribution of the property for the events in unaffected children.

Supplementary References

1 Yoon, S., Xuan, Z., Makarov, V., Ye, K. & Sebat, J. Sensitive and accurate detection of copy number variants using read depth of coverage. *Genome Res* **19**, 1586-1592, doi:10.1101/gr.092981.109 (2009).

2 Durbin, R. *Biological sequence analysis : probabalistic models of proteins and nucleic acids*. (Cambridge University Press, 1998).

3 Neuhäuser, M. in *International Encyclopedia of Statistical Science* (ed Miodrag Lovric) 1656-1658 (Springer Berlin Heidelberg, 2011).

4 Siepel, A. *et al.* Evolutionarily conserved elements in vertebrate, insect, worm, and yeast genomes. *Genome Res* **15**, 1034-1050, doi:10.1101/gr.3715005 (2005).

5 Cooper, G. M. *et al.* Distribution and intensity of constraint in mammalian genomic sequence. *Genome Res* **15**, 901-913, doi:10.1101/gr.3577405 (2005).

6 Kircher, M. *et al.* A general framework for estimating the relative pathogenicity of human genetic variants. *Nat Genet* **46**, 310-315, doi:10.1038/ng.2892 (2014).

7 O'Leary, N. A. *et al.* Reference sequence (RefSeq) database at NCBI: current status, taxonomic expansion, and functional annotation. *Nucleic Acids Res* **44**, D733-745, doi:10.1093/nar/gkv1189 (2016).

8 Jaganathan, K. *et al.* Predicting Splicing from Primary Sequence with Deep Learning. *Cell* **176**, 535-548 e524, doi:10.1016/j.cell.2018.12.015 (2019).

9 Kong, A. *et al.* Rate of de novo mutations and the importance of father's age to disease risk. *Nature* **488**, 471-475, doi:10.1038/nature11396 (2012).

10 Wong, W. S. *et al.* New observations on maternal age effect on germline de novo mutations. *Nat Commun* **7**, 10486, doi:10.1038/ncomms10486 (2016).

11 Goldmann, J. M. *et al.* Parent-of-origin-specific signatures of de novo mutations. *Nat Genet* **48**, 935-939, doi:10.1038/ng.3597 (2016).

12 Iossifov, I. *et al.* De novo gene disruptions in children on the autistic spectrum. *Neuron* **74**, 285-299, doi:10.1016/j.neuron.2012.04.009 (2012).

13 in *Encyclopedia of Biometrics* (eds Stan Z. Li & Anil Jain) 1376-1376 (Springer US, 2009).
