# Supplementary material for: Rates of contributory de novo mutation in high and low-risk autism families
Source: Commun Biol. 2021 Sep 1;4:1026. doi: 10.1038/s42003-021-02533-z (PMC8410909; doi:10.1038/s42003-021-02533-z)
Supplement: Supplementary file 2 — Description of Additional Supplementary Files [file 42003_2021_2533_MOESM2_ESM.pdf]

Description of Additional Supplementary Files

File name: Supplementary Data 1

Description: The table lists all the children from the SSC and AGRE collections that are part of this study, including the children that we determined to have excessive cell-line genomic drift indicated in the ‘cell line drift outlier’ column.

File name: Supplementary Data 2

Description: The table lists the small-scale de novo variants identified in the children of the SSC that pass the cell-line genetic drift filters.

File name: Supplementary Data 3

Description: The table lists the small-scale de novo variants identified in the children of the AGRE collection that pass the cell-line genetic drift filters.

File name: Supplementary Data 4

Description: The table lists the de novo CNV variants identified in the children of SSC and AGRE collections that pass the cell-line genetic drift filters.

File name: Supplementary Data 5

Description: The table lists all protein-coding genes from RefSeq and the numbers of de novo variants of six categories listed in the Denovo-db repository. The “number of autism LGD variants,” “number of autism missense variants,” and “number of autism synonymous variants” columns show the number of de novo LGD, missense, and synonymous variants, respectively, identified in children with autism. The “number of all NDD LGD variants,” “number of all NDD missense variants,” “number of all NDD synonymous variants” similarly show the number of de novo variants of the three types identified in children diagnosed with one of the following neurodevelopmental disorders (NDD): autism, schizophrenia, intellectual disability, developmental delay, or epilepsy.

File name: Supplementary Data 6

Description: The table lists all de novo candidates generated by the two methods for identifying de novo CNVs, the EWT de novo CNV finder and the HMM de novo CNV finder before the stringent population and cell-line genetic drift filter are applied. It is included

here to assist the reader interested in getting a deeper understanding in our methods for identifying, merging, and filtering de novo CNVs.

File name: Supplementary Data 7

Description: The table lists the de novo CNV events reported in Sanders, et al. in the SSC samples we examined in this work but that were not included in our analysis due to our stringent filters. For each of these excluded events we provide an explanation of why we have excluded it. Only a small number (6) of events were not detected at in our data. The rest were excluded because of mosaicism detected in parents, overlap with polymorphic CNVs, or because the child happened to fail our cell-line genetic drift filters.
