# Supplementary material for: Rates of contributory de novo mutation in high and low-risk autism families
Source: Commun Biol. 2021 Sep 1;4:1026. doi: 10.1038/s42003-021-02533-z (PMC8410909; doi:10.1038/s42003-021-02533-z)
Supplement: Supplementary file 10 — Reporting Summary [file 42003_2021_2533_MOESM10_ESM.pdf]

nature research

Corresponding author(s): Ivan Iossifov

Last updated by author(s): 7/22/2021

Reporting Summary

Nature Research wishes to improve the reproducibility of the work that we publish. This form provides structure for consistency and transparency in reporting. For further information on Nature Research policies, see our Editorial Policies and the Editorial Policy Checklist.

Statistics

For all statistical analyses, confirm that the following items are present in the figure legend, table legend, main text, or Methods section.

n/a	Confirmed	
☐	☒	The exact sample size (*n*) for each experimental group/condition, given as a discrete number and unit of measurement
☒	☐	A statement on whether measurements were taken from distinct samples or whether the same sample was measured repeatedly
☐	☒	The statistical test(s) used AND whether they are one- or two-sided<br>*Only common tests should be described solely by name; describe more complex techniques in the Methods section.*
☐	☒	A description of all covariates tested
☐	☒	A description of any assumptions or corrections, such as tests of normality and adjustment for multiple comparisons
☐	☒	A full description of the statistical parameters including central tendency (e.g. means) or other basic estimates (e.g. regression coefficient) AND variation (e.g. standard deviation) or associated estimates of uncertainty (e.g. confidence intervals)
☐	☒	For null hypothesis testing, the test statistic (e.g. *F*, *t*, *r*) with confidence intervals, effect sizes, degrees of freedom and *P* value noted<br>*Give P values as exact values whenever suitable.*
☒	☐	For Bayesian analysis, information on the choice of priors and Markov chain Monte Carlo settings
☒	☐	For hierarchical and complex designs, identification of the appropriate level for tests and full reporting of outcomes
☐	☒	Estimates of effect sizes (e.g. Cohen's *d*, Pearson's *r*), indicating how they were calculated

Our web collection on statistics for biologists contains articles on many of the points above.

Software and code

Policy information about availability of computer code

Data collection	We used a custom pipeline to identify de novo variants from preexisting whole-genome sequencing data stored in bam files. We describe the pipeline in detail in the Methods and Supplementary Note 1.
Data analysis	The source of the scripts we used in our analysis is deposited in the public GitHub repository https://github.com/iossifovlab/denovoInHighAndLowRiskPaper. The python scripts use input only from Supplementary Data 1-5 and completely re-produce all the results discussed in the manuscript.

For manuscripts utilizing custom algorithms or software that are central to the research but not yet described in published literature, software must be made available to editors and reviewers. We strongly encourage code deposition in a community repository (e.g. GitHub). See the Nature Research guidelines for submitting code & software for further information.

Data

Policy information about availability of data

All manuscripts must include a data availability statement. This statement should provide the following information, where applicable:

- Accession codes, unique identifiers, or web links for publicly available datasets
- A list of figures that have associated raw data
- A description of any restrictions on data availability

We obtained existing whole-genome sequence datasets for the Simons Simplex Collection (SSC) and the AGRE collections that have already been used in published manuscripts. The SSC sequence data can be obtained from Simons Foundation Research Initiative (SFARI; https://www.sfari.org). The AGRE sequence data can be obtained from the Hartwell Foundation's Autism Research and Technology Initiative (iHART; http://www.ihart.org). Access to these resources is subject to approval by the respective institutions. We used these whole-genome sequencing data to identify de novo substitutions, de novo small indels, and de novo CNV using the

methods described above and in Supplementary Note 1. We provide the complete lists of children and identified de novo variants together with all attributes used in our analysis in Supplementary Data 1-5.

Field-specific reporting

Please select the one below that is the best fit for your research. If you are not sure, read the appropriate sections before making your selection.

☒ Life sciences ☐ Behavioural & social sciences ☐ Ecological, evolutionary & environmental sciences

For a reference copy of the document with all sections, see nature.com/documents/nr-reporting-summary-flat.pdf

Life sciences study design

All studies must disclose on these points even when the disclosure is negative.

Sample size	We used all the available whole-genome sequence data from the two family collections related to autism, SSC and AGRE.
Data exclusions	We excluded few samples during sequence-related quality control. We also excluded samples that exhibited excessive cell-line genetic drift, as described in the Results.
Replication	We did not replicate the findings in separate datasets. Unfortunately, there was no available autism family whole-genome data of sufficient size for meaningful replication at the time of writing.
Randomization	This study is an observational one, and thus we did not perform randomization.
Blinding	The processing of the samples was done uniformly across the study groups by a computational pipeline. In addition, we performed appropriate normalization to control factors (i.e., sequencing depth and parental ages) that could influence our comparisons of rates of de novo variants. We describe the details of normalization procedures in the Results.

Reporting for specific materials, systems and methods

We require information from authors about some types of materials, experimental systems and methods used in many studies. Here, indicate whether each material, system or method listed is relevant to your study. If you are not sure if a list item applies to your research, read the appropriate section before selecting a response.

Materials & experimental systems

n/a	Involved in the study	
☒	☐	Antibodies
☒	☐	Eukaryotic cell lines
☒	☐	Palaeontology and archaeology
☒	☐	Animals and other organisms
☒	☐	Human research participants
☒	☐	Clinical data
☒	☐	Dual use research of concern

Methods

n/a	Involved in the study	
☒	☐	ChIP-seq
☒	☐	Flow cytometry
☒	☐	MRI-based neuroimaging
